# Informal risk-sharing between smallholders may be threatened by formal insurance: Lessons from a stylized agent-based model

**DOI:** 10.1371/journal.pone.0248757

**Published:** 2021-03-19

**Authors:** Meike Will, Jürgen Groeneveld, Karin Frank, Birgit Müller

**Affiliations:** 1 Department of Ecological Modelling, Helmholtz Centre for Environmental Research – UFZ, Leipzig, Germany; 2 German Centre for Integrative Biodiversity Research (iDiv) Halle-Jena-Leipzig, Leipzig, Germany; 3 Institute of Environmental Systems Research (IUSF), University of Osnabrück, Osnabrück, Germany; Neijiang Normal University, CHINA

## Abstract

Microinsurance is promoted as a valuable instrument for low-income households to buffer financial losses due to health or climate-related risks. However, apart from direct positive effects, such formal insurance schemes can have unintended side effects when insured households lower their contribution to traditional informal arrangements where risk is shared through private monetary support. Using a stylized agent-based model, we assess impacts of microinsurance on the resilience of those smallholders in a social network who cannot afford this financial instrument. We explicitly include the decision behavior regarding informal transfers. We find that the introduction of formal insurance can have negative side effects even if insured households are willing to contribute to informal risk arrangements. However, when many households are simultaneously affected by a shock, e.g. by droughts or floods, formal insurance is a valuable addition to informal risk-sharing. By explicitly taking into account long-term effects of short-term transfer decisions, our study allows to complement existing empirical research. The model results underline that new insurance programs have to be developed in close alignment with established risk-coping instruments. Only then can they be effective without weakening functioning aspects of informal risk management, which could lead to increased poverty.

## Introduction

Within its Sustainable Development Goals, the United Nations has identified the eradication of poverty as one of the most important goals that humanity should meet by 2030 [1]. An essential contribution to achieving this target is to ensure that vulnerable households are effectively protected against extreme climate-related events and other economic, social and ecological shocks and disasters [2]. Traditionally, households in rural communities across the world manage to cope with such threats through informal arrangements [3, 4]. These risk-sharing networks buffer income shocks by an exchange of money, labor or in-kind goods between households in need and those with the capacity to help. Various forms of such informal networks exist. In Ethiopia, for example, group-based support arrangements with often hundreds of members, so-called *iddirs*, offer informal insurance to compensate costs for funerals, medical expenses or food shortage against the payment of a premium [5–7]. Similarly, in Burkina Faso, individuals linked by neighborship, religious confession or shared ethnic affiliation arrange in *tontines* and make monetary contributions to a common fund from which risk-sharing is financed [8]. Among the Maasai, the bilateral gift-giving concept *osotua* is established where, based on reciprocity, households exchange livestock or other goods when in need [9]. However, when the whole risk-sharing network is affected by a large-scale extreme event such that many households suffer substantial losses simultaneously, private transfers can no longer provide buffering [10, 11]. Droughts or floods, which both are expected to increase under climate change [12–15], are an example of such shocks. Insurance products specifically designed for the needs of low-income households in developing countries, known as microinsurance or inclusive insurance, are seen as an effective tool to address these challenges and are therefore highly promoted and supported by governments in recent years. Current programs to help vulnerable countries, particularly in the southern hemisphere, include the G7 ‘InsuResilience’ initiative launched in 2015 [16] or the Global Index Insurance Facility managed by the World Bank Group [17].

However, apart from direct positive effects, the introduction of formal insurance in communities where informal risk-coping instruments exist may have unintended side effects [18]. In lab-in-the-field experiments and household surveys covering different cultural contexts and insurance products, evidence has been found that formal insurance can crowd-out informal risk-sharing arrangements [19–24].

It was shown that households reduce their willingness to provide informal support if they themselves do not need any other risk coverage apart from insurance. In the case of index insurance in Ethiopia, the results of household surveys suggest, conversely, that the availability of insurance could encourage informal transfers, as insured households are better able to help [25, 26]. Theoretical models show, on the one hand, that the introduction of insurance can lead to a decline in welfare due to reduced private transfers [27, 28], but also that informal safety nets and microinsurance can complement each other in the presence of basis risk—the potential mismatch between actual losses and received insurance payouts [7, 29].

This broad range of studies underlines the different implications that the introduction of formal insurance can have on people’s behavior towards informal transfers. However, long-term effects of these changes on the resilience of low-income households, particularly through a direct comparison of the various potential behavioral responses to private monetary support, have not yet been investigated. To address this gap, we develop an agent-based model that considers smallholders in a social network and captures dynamics between income losses, insurance payments and informal risk-sharing. We focus our analysis on smallholder farming, a predominant form of rural agriculture in developing countries that is driven by subsistence production. Households practicing this type of agriculture have limited financial means to deal with the multiple risks that affect them individually or hit an entire community. Therefore, they rely on effective mechanisms to cope with risk [30]. By using an agent-based modelling approach, we exploit several advantages compared to empirical and theoretical methods already applied in studies on formal and informal insurance. First, insights can be gained independently of the specific local context and where empirical data is lacking [31, 32]. In contrast to household surveys or behavioral games that cover only short time spans limited to specific regions, a model can represent conditions from several regions with different risk contexts independent of a particular case study. Second, agent-based models allow us to include complex strategies of human decision making [33, 34] that go beyond economic rationales implemented in existing theoretical models on formal and informal insurance. In particular, it is possible to integrate that households differ in their behavior as they adapt their decisions to individual characteristics and influences from their environment [35]. Third, with our model we can analyze the implications of transfer behavior of households linked to several others in a network. In most theoretical models, it is assumed that households interact only bilaterally or that all households in a community are connected. However, when households have a limited number of neighbors that they can request for help, this can give crucial information on how effective monetary transfers can be. In the context of informal risk-sharing, agent-based modelling has already helped to assess whether traditional gift-giving relationships increase the viability of pastoralists’ herds [36, 37] and how spatial and temporal correlations of shock events impact the resilience of households [38]. Furthermore, agent-based models have been used to analyze the ecological effects of formal insurance on rangeland management and pasture conditions [39, 40].

With our study, we contribute to that research strain by evaluating impacts of the combination of formal and informal risk-sharing mechanisms. The main objective of our model is to reveal unintended social consequences of insurance programs when households additionally help each other informally when in need. Specifically, we analyze whether and how economic needs of households (i.e. level of living costs) and characteristics of extreme events (i.e. frequency, intensity and type of shock) influence the ability of formal insurance and informal risk-sharing to buffer income losses. We assume that households are connected in a social network and can request money from their neighbors when their financial resources are not sufficient to sustain themselves. We explicitly distinguish two types of behavior with regard to monetary transfers that are based on observations from empirical studies. First, we assume that all households provide financial resources whenever they are requested and can afford to (solidarity). Second, we simulate scenarios where only uninsured households show solidarity and insured households do not transfer (no solidarity). With its stylized characterization of transfer behavior and budget dynamics, our modelling approach provides a qualitative understanding of when formal insurance complements existing risk mitigation tools and when potentially reduced support from insured households has harmful consequences for the resilience of smallholders. On the basis of a systematic analysis of external conditions and human behavior, we highlight aspects that are necessary for effective insurance design to prevent a degradation of functioning aspects of informal risk management and thus avoid an increase in poverty.

## Methods

### Model description

The model is not used to analyze a particular case study, but represents conditions from several regions with different risk contexts where informal support networks between smallholder farmers are prevalent. We simulate *N*_H_ = 50 households which roughly corresponds with empirical observations of traditional support arrangements [6–8]. Each agent *H*_*i*_, *i* = 1, …, *N*_H_, represents a smallholder household and is characterized by its budget *Y*_*i*_. Households are endowed with an initial budget *Y*^0^. They generate a regular yearly income *I* and have to spend an amount *C* to cover annual living costs. The population is homogeneous with all households having the same initial budget, income level and annual living costs. Income shocks reduce the budget of a household by an amount *S* if the household is affected. We distinguish unexpected events which are either idiosyncratic, hitting the households independently (such as illness), or covariate, affecting many households at the same time (such as droughts or floods). Only one shock type is considered per simulation run. Idiosyncratic shocks occur with a probability *p*_s_ for each household. For covariate shocks, the chance of a shock at village level is *p*_V_. If such a shock occurs, households are hit with probability *p*_H_. Households that are not affected in this case might, for example, have a more favorable geographical location in case of floods or an agricultural management strategy more adapted to drought risks. Overall, this results in a shock probability *p*_s_ = *p*_V_ × *p*_H_ for an individual household.

To smooth income shocks households can engage in informal safety nets. Households are connected in an undirected network on which they can request money from and donate money to other households. The network is imposed during the initialization of the model and is kept constant (i.e. static) for a simulation run. We have implemented small-world networks using the Watts-Strogatz model [41]. This algorithm creates a regular ring network with each household connected to *N*_N_/2 neighbors on either side and each link rewired with probability *p*_r_.

Some households have access to formal insurance schemes. Why households decide to insure is currently a highly explored topic. Next to purely economic aspects, social and cultural influences such as risk aversion and influence from peers or personal and demographic factors such as age and gender are also considered being important [4, 42]. Explicitly including reasons behind the decision to insure is therefore out of the scope of this paper. Hence, we assume that a fixed proportion *γ* of households is informed about insurance and choose to buy it. Insurance status is then randomly assigned to households at the beginning of the simulation and is kept throughout the simulated period. Insured households insure their complete income. We model indemnity insurance that covers the actual losses a household suffers from. The payout *α* in case of a shock is *α* = *S*. The yearly premium *β*, which insured households have to pay, is actuarially fair and thus equals the expected loss given the shock probability *p*_s_ and the shock intensity *S* and reads *β* = *p*_s_ × *S*.

Each household’s objective is to maintain prosperity with a budget above or equal to zero. Households whose budget is below this threshold may receive transfers from households with whom they share a link in the network that are rich enough to help others, i.e. that have a budget above zero. The household randomly picks one of its neighbors and requests transfers. If the request cannot be fulfilled by one single agent, households continue requesting the missing amount from other agents in their network. We explicitly distinguish two types of transfer behavior: solidarity and no solidarity. For simplicity, in one simulation run all households decide on their transfers according to the same strategy. When households show solidarity, they transfer whenever they can afford it. This implies that households may assume that the requesting household will return the transfer in the future if they need support themselves. Since, in the simulated scenarios, insurance covers all losses, this will only occur for uninsured households. It is incorporated that donors do not put themselves at financial risk through transfers. Therefore, it is ensured that the minimum budget of a donor after a transfer is zero. On the other hand, the household in need should not get too rich through the help of others. The maximum achievable budget through support of other households is thus also zero. For the second type of transfers (no solidarity), only uninsured households show solidarity and contribute to informal risk-sharing whenever they can afford it; insured households do not transfer at all. Here, we assume that insured households refuse contribution as they are not dependent on reciprocal behavior of others.

We assume that if households do not manage to reach the poverty threshold either on their own or with the support of their neighbors, they must leave the system. This implicitly includes that households that cannot cover their living costs may migrate to other regions where they expect to strengthen their resilience to shocks through improved economic, environmental or social conditions [43, 44] but neglects that households may have other sources of support from outside the village that they could use to cover their losses [45, 46]. We condense the capacity of households to cope with income shocks in a ‘survival rate’ that indicates the fraction of households that manages to maintain a budget above zero over the simulated time span.

In order to make our observations comparable between scenarios with varying number of insured households, we present the results always for the same subgroup of households. When referring to uninsured households, we determined the reference group by all households that are uninsured in the case with the highest insurance rate (*N*_H_ = 20, *γ* = 60%). We ensured that these households are uninsured in every other scenario. The shock exposure, network relationships and transfer requests of this reference group is the same for each repetition of the simulation run regardless of the specific scenario. When presenting results for insured households, we refer to those households that are insured in the scenario with lowest insurance rate (*N*_H_ = 15, *γ* = 30%). These households are insured in every scenario (except *γ* = 0%).

The model uses discrete annual time steps and a long-term perspective of *T* = 50 years is assumed. For each setting, we have carried out 100 repetitions. A detailed model description in a structured form based on the ODD+D protocol [47] can be found in S1 Appendix. The model is implemented in NetLogo and available to download at CoMSES Net [48].

### Parameter selection

We calculate the expected value of budget change per time step to select parameter combinations for living costs *C*, shock probability *p*_s_ and shock intensity *S* in a range where formal and informal insurance can both be used effectively. This implies (1) that the shock intensity should be high enough to make financial protection necessary and (2) that formal insurance should be affordable. Additionally to the mathematical restrictions, we constrain the parameters with respect to ecological and economic observations. We divide all parameter ranges in equidistant steps of 0.1, which results in 52 reasonable parameter combinations that meet the constraints. A more detailed description of the selection procedure can be found in S2 Appendix.

## Results

### Effectiveness of risk-coping instruments over time

To illustrate the effectiveness of different risk-coping instruments, we present simulation runs over 50 years for one specific parameter combination of income *I*, living costs *C*, shock probability *p*_s_ and shock intensity *S* (*I* = 1, *C* = 0.8, *p*_s_ = 0.3, *S* = 0.6; *I* is normalized to 1, *C* and *S* are unitless and related to *I*). We consider idiosyncratic shocks and analyze the results for a small-world risk-sharing network with rewiring probability *p*_r_ = 0.2 and an average number of four neighbors (*N*_N_ = 4). Results for a more random network (*p*_r_ = 0.8) and more (*N*_N_ = 8) or less (*N*_N_ = 2) neighbors can be found in S3 Appendix. Our analysis covers different risk-coping scenarios depending on the availability of insurance and informal transfers and the transfer decision of insured households. We assume three main types of transfer behavior: (1) no informal transfer, (2) all households show solidarity and (3) only uninsured households show solidarity. Additionally, we distinguish three levels of insurance rates *γ* all households are uninsured (*γ* = 0%), a small part (*γ* = 30%) and a large part (*γ* = 60%) of households is insured.

**Fig 1:**
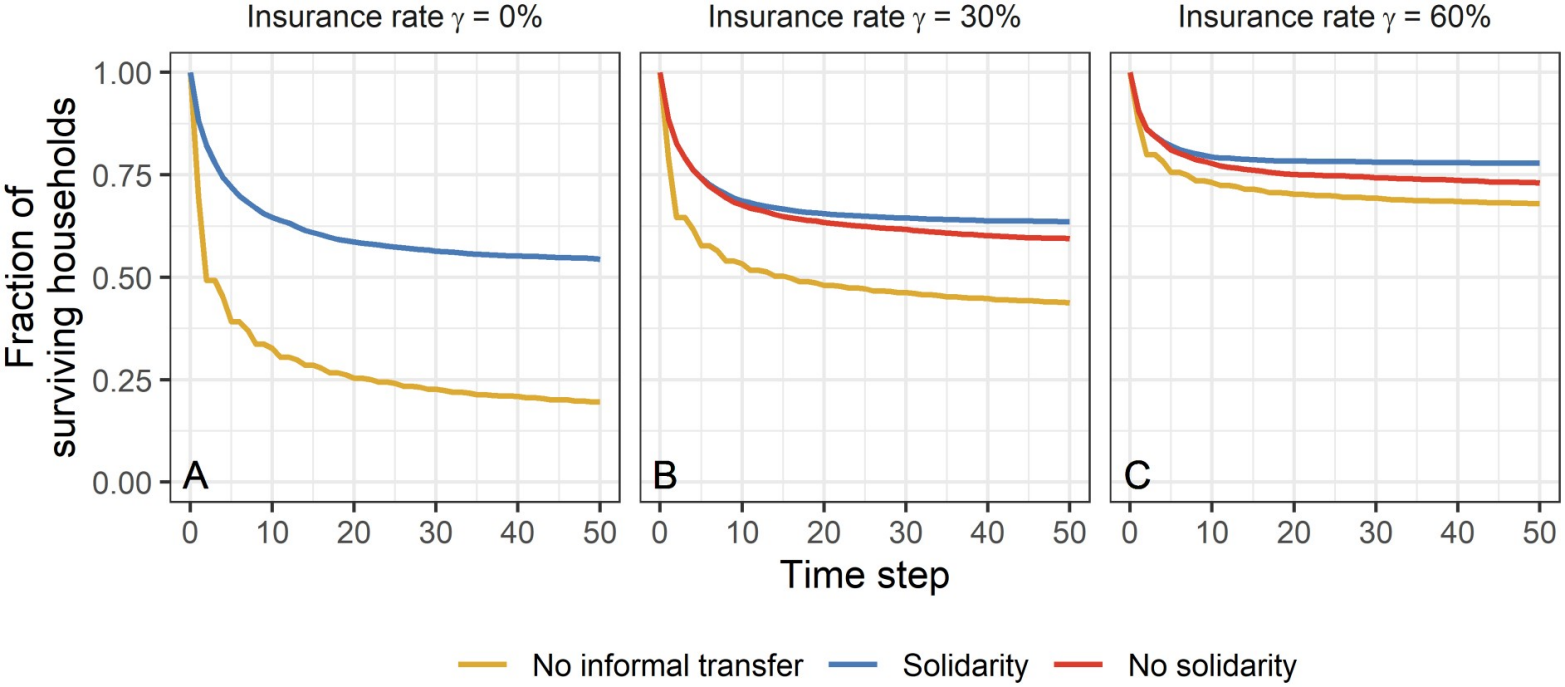
Fraction of surviving households. Fraction of surviving households for different risk-coping instruments and insurance rates *γ* (A—0%, B—30%, C—60%). Results show the mean over 100 repetitions for a selected parameter combination of income *I*, living costs *C*, shock probability *p*_s_ and shock intensity *S* (*I* = 1, *C* = 0.8, *p*_s_ = 0.3, *S* = 0.6).

**Fig 2:**
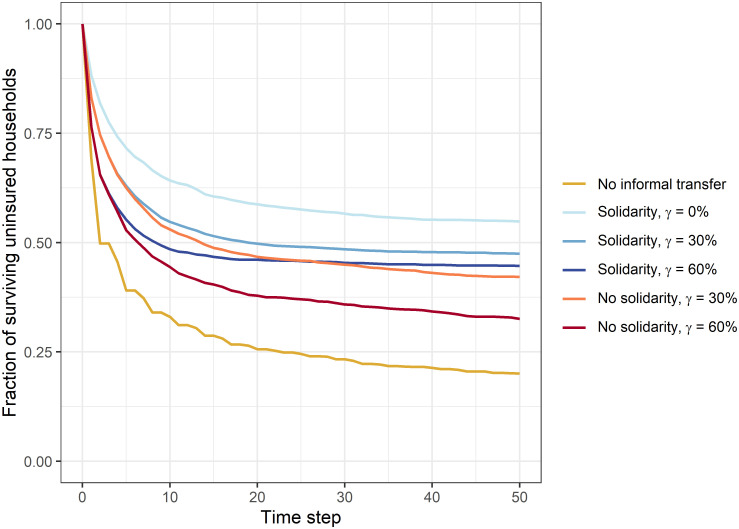
Fraction of surviving uninsured households. Fraction of surviving uninsured households among the 20 households that are uninsured in every scenario for different risk-coping instruments with insurance rates *γ*. Results show the mean over 100 repetitions for a selected parameter combination of income *I*, living costs *C*, shock probability *p*_s_ and shock intensity *S* (*I* = 1, *C* = 0.8, *p*_s_ = 0.3, *S* = 0.6). Without informal transfers the survival rates are independent of the insurance rates and the resulting curves for different insurance rates would overlap. They are therefore not represented separately.

When considering the fraction of surviving households for different risk-coping instruments and insurance rates (Fig 1), we observe that informal transfers, independent of the transfer decision, have a positive impact on the survival rates. To disentangle the effects of insurance and decisions behind informal transfers, we have separately investigated the survival rates for uninsured households. In order to make our observations comparable between scenarios with varying number of insured households, we present the results always for the same subgroup of households. The survival rate of uninsured households is lower when insurance is available than when households cover their risks only through informal risk-sharing (Fig 2). For the selected external conditions, the introduction of insurance thus has negative effects for uninsured households. Even if insured households maintain showing solidarity, the introduction of insurance diminishes the survival rate of uninsured households: Shortly after the introduction the same number of uninsured households has to leave the system as if insured households refuse to contribute to informal transfers. Only in the long run, solidarity of insured households has a positive effect on uninsured households.

**Fig 3:**
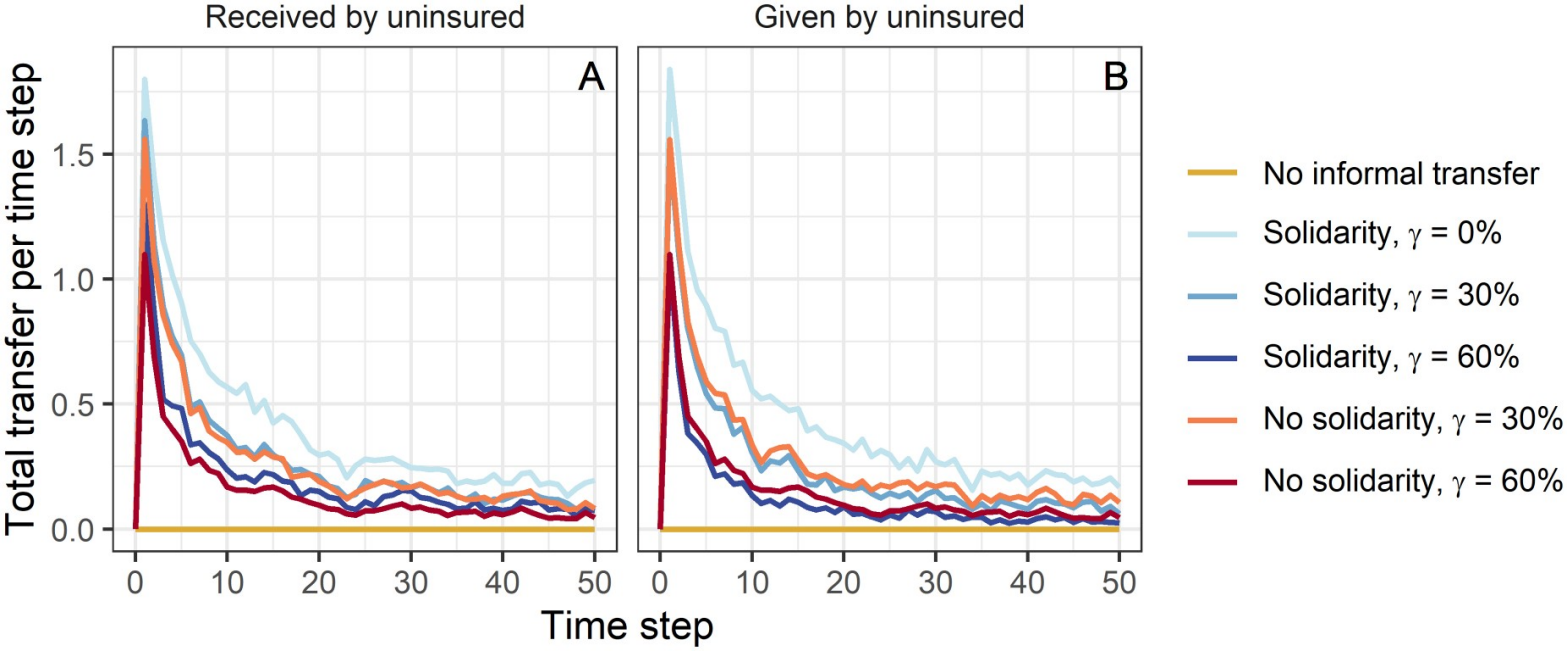
Total transfer. Total transfer (A) received and (B) given by all 20 households that are uninsured in every scenario per time step. Results show the mean over 100 repetitions for a selected parameter combination of income *I*, living costs *C*, shock probability *p*_s_ and shock intensity *S* (*I* = 1, *C* = 0.8, *p*_s_ = 0.3, *S* = 0.6).

This can be explained by the total transfer that the selected uninsured households have given and received per time step (Fig 3). When more households cover their risks with formal insurance, less households need to request informal transfers. For these circumstances, our model results indicate that the transfer amount is lower when insurance rates are higher. However, the selected households receive less transfers also due to the lower contributions by insured households (Fig 3A). In the first time step, the transfer demand of the selected households is equal regardless of the scenario (not shown here). Each uninsured household affected by a shock needs support. However, even if insured households were in general willing to help, the total transfer was lower than in the case without insurance. From this it can be concluded that insured households did not contribute as much as uninsured households. Due to the premium payments, which lower their available budget, their ability to help through informal transfers is weakened. Especially shortly after the introduction of insurance, where insured households have not benefited much from refunded losses, this reduces the number of surviving uninsured households. Furthermore, we can see that, if insured households do not show solidarity, the side effects of the introduction of insurance is even worse. Not only do uninsured households receive less, they also have to transfer more compared to when insured households contribute as well (Fig 3B). This is particularly evident for high insurance rates. If transfers are only provided by households that are themselves vulnerable to transfers, this in the end lowers their own ability to cope with future losses and leads to even lower survival rates.

**Fig 4:**
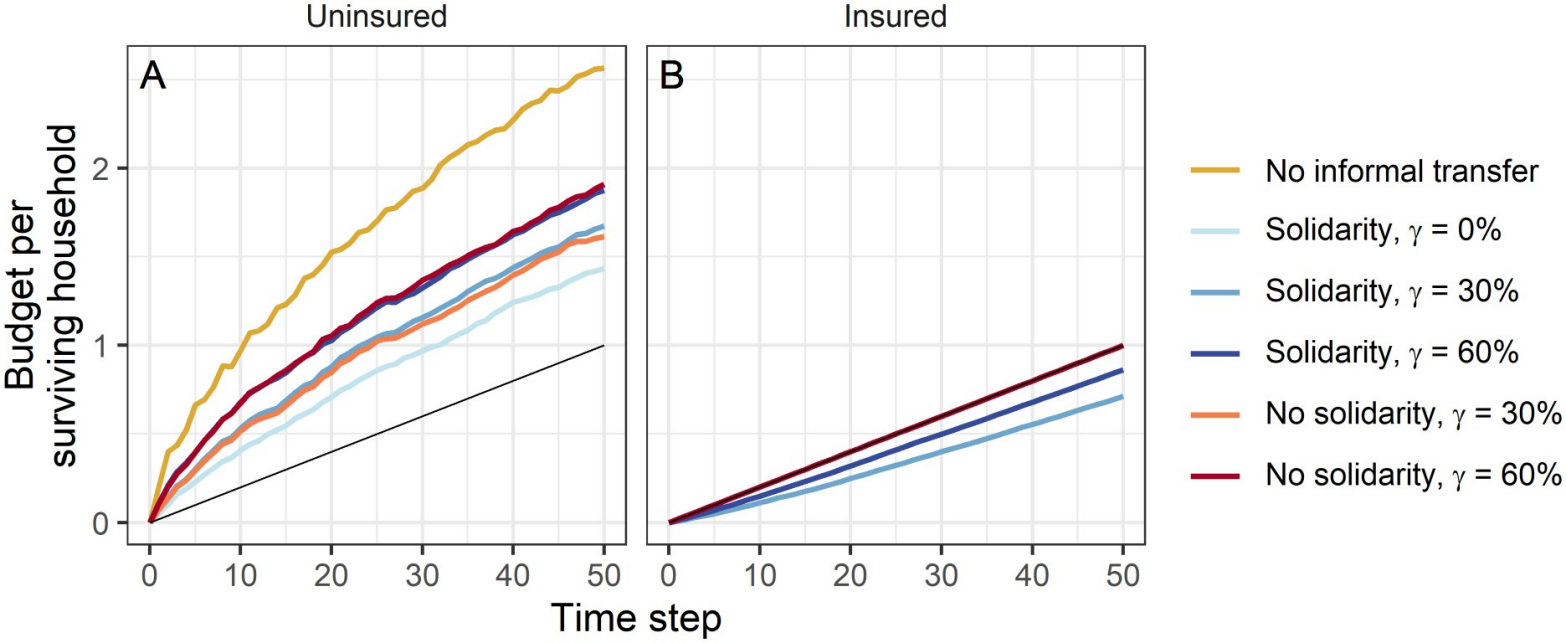
Budget per surviving household. Budget per surviving household calculated based on (A) the 20 households that are uninsured in every scenario and (B) the 15 households that are insured in every scenario (except *γ* = 0%). The straight line shows the maximum budget that an insured household can receive as reference value. Results show the mean over 100 repetitions for a selected parameter combination of income *I*, living costs *C*, shock probability *p*_s_ and shock intensity *S* (*I* = 1, *C* = 0.8, *p*_s_ = 0.3, *S* = 0.6). Without informal transfers the budget per surviving household is independent of the insurance rates and the resulting curves for different insurance rates would overlap. They are therefore not represented separately.

Resilience to shocks, i.e. the ability to recover from losses, is manifested not only in whether households survive at all but also in the amount of their financial resources. By comparing the budgets of the surviving insured and uninsured households separately (Fig 4), we can observe financial consequences of insurance and informal transfers. This may help to understand reasons for the empirically observed transfer decisions of insured households. Comparing the budgets of the surviving households underlines that individual uninsured households manage to obtain substantially higher budgets than insured households (Fig 4A). However, especially in the scenario without informal transfers, this applies only to a small fraction of households and can therefore not be seen as a sustainable strategy to ensure that budgets are resilient to shocks. Additionally, since the transfers are not repaid, these gains are at the expense of the insured households that show solidarity, which end up with a budget that is lower than what they could have received without helping households in need (Fig 4B).

For all outcome measures, we observe differences between the scenarios with and without solidarity of insured households. As showing no solidarity de facto reduces the links of the network that embeds the uninsured households, this indicates the importance of the number of neighbors. To investigate the relevance of the network structure more systematically, we present the same outcome measures for a small-world network with more (*N*_N_ = 8) or less (*N*_N_ = 2) neighbors in S3 Appendix. Overall, these results confirm our hypothesis and underline that a network with more interactions leads to higher resilience of uninsured households.

### Effectiveness of risk-coping instruments for different external conditions

**Fig 5:**
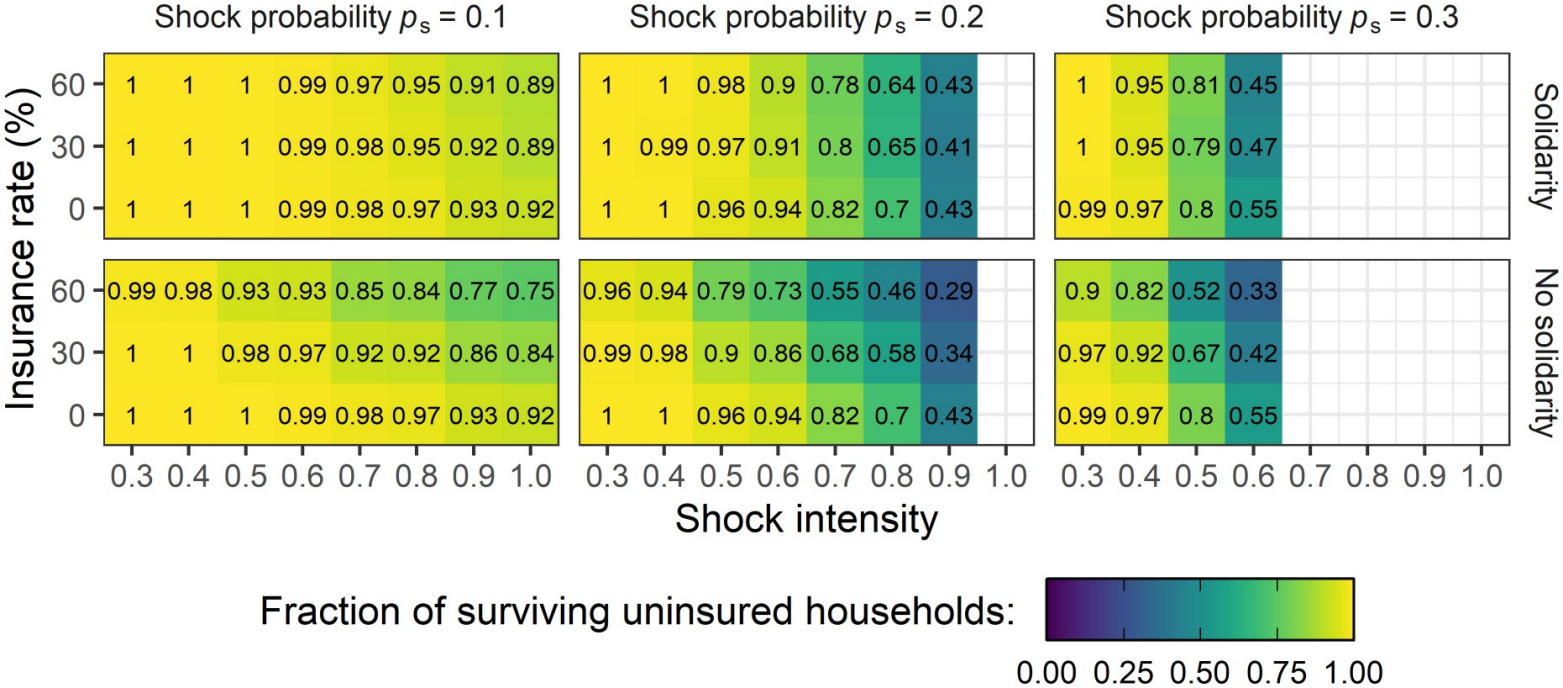
Idiosyncratic shocks. Fraction of surviving uninsured households among the 20 households that are uninsured in every scenario depending on insurance rates *γ*, shock probability *p*_s_ and shock intensity *S* for idiosyncratic shocks with fixed income (*I* = 1) and level of living costs (*C* = 0.8). Upper rows show the results for solidarity between all households, lower rows show the results for solidarity between uninsured households only. The darker the color the less households survive, numbers in each panel denote the exact fraction. If a panel is left blank, the parameter combination is economically not feasible (see S2 Appendix) and therefore not selected for the analysis. Results show the mean over 100 repetitions of the number of surviving uninsured households at the last simulation step (*t* = 50).

So far, we focused on the consideration of an exemplary scenario of external conditions. To investigate the transferability of these observations to a broader range of living costs and increased or decreased shock probability as well as intensity in a systematic way, we analyzed the behavior of the system for all parameter combinations that were found to be economically feasible (see S2 Appendix for the selection criteria). We compared the effects of 50 years of purely informal transfers (*γ* = 0%) on the survival rate of uninsured households to the situation 50 years after the introduction of insurance with low (*γ* = 30%) and high (*γ* = 60%) insurance rates, respectively. In Fig 5, we present the model results for a fixed income (*I* = 1) and a fixed level of living costs (*C* = 0.8). Results for higher and lower annual expenses and different network structures can be found in S4 Appendix. In general, we see that for more severe shocks, i.e. higher shock intensity, less uninsured households survive. For the same external conditions, in many cases the respective survival rate of uninsured households is lower if a fraction of households is insured, even if after the introduction of insurance all households show solidarity and contribute to informal transfers. This is due to the premium payments that lead to missing budget of insured households to help others. For few cases, there is no clear effect of the introduction of insurance with prevailing solidarity. For these external conditions, uninsured households are not harmed by the introduction of insurance but they do not benefit either. If insured households are no longer willing to help uninsured households in need, there is a clear trend that this leads to lower survival rates among uninsured households. In this case, the informal support has to be covered by a smaller subgroup of households that are willing to transfer. This lowers the available budget of these households and might bring more uninsured households to financially critical situations. In addition, especially for high insurance rates, the network of households willing to participate in transfer arrangements is thinned out. Hence, households in need may no longer be connected to households willing to help them.

The overall conclusions drawn from the selected parameter combination are therefore found to be robust and valid for a broad range of external conditions with different levels of living costs, probabilities for shock occurrence and shock intensity. Households that cannot afford formal insurance do not benefit from its introduction even if their insured peers are willing to help them. In many situations, insured households might simply be not able to cover requests from the informal network in addition to their regular premium payments.

### Effectiveness of risk-coping instruments for covariate shocks

**Fig 6:**
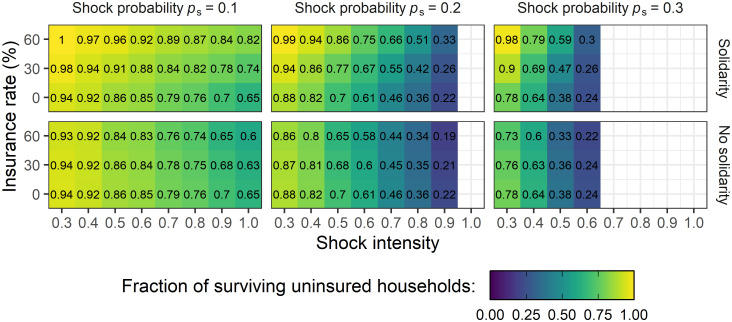
Covariate shocks. Fraction of surviving uninsured households among the 20 households that are uninsured in every scenario depending on insurance rates *γ*, shock probability *p*_s_ and shock intensity *S* for covariate shocks with fixed income (*I* = 1) and level of living costs (*C* = 0.8). In case of a shock at village level, 80% of the households are affected (*p*_H_ = 0.8). Upper rows show the results for solidarity between all households, lower rows show the results for solidarity between uninsured households only. The darker the color the less households survive, numbers in each panel denote the exact fraction. If a panel is left blank, the parameter combination is economically not feasible (see S2 Appendix) and therefore not selected for the analysis. Results show the mean over 100 repetitions of the number of surviving uninsured households at the last simulation step (*t* = 50).

To investigate how shocks which affect many households simultaneously stress the performance of informal risk-coping instruments, we conduct the same model analysis for covariate shocks. We again present an overview of the behavior of the system for all economically feasible parameter combinations and conduct the analysis for the subset of those households that are uninsured in every scenario to allow for best comparison. In Fig 6, we show the resulting survival rates of uninsured households when 80% of the households are affected if a shock hits the village (*p*_H_ = 0.8). The model results for the more extreme case in which all households are affected by the shock (*p*_H_ = 1) can be found in S5 Appendix. Although, in total, each household suffers equally often from a shock in the idiosyncratic and the covariate cases, the survival rate of uninsured households is lower when they are exposed to covariate shocks in all external conditions which were considered. This implies that protection against this type of shock is more difficult without formal insurance. In contrast to what we observed for idiosyncratic shocks, the introduction of insurance leads to slightly higher survival rates of uninsured households if insured households are willing to contribute informal transfers. This is because, in case of idiosyncratic shocks, uninsured households were in general able to make larger contributions to the transfers than insured households if not in need themselves. For covariate shocks, however, it is unlikely that an uninsured neighbor is able to make a contribution at all, as many households are in need at the same time. In this case, even the sometimes small contribution of insured households can help to ensure the survival of some uninsured households. On the other hand, if insured households are not willing to give transfer payments, this leads, as in the case of idiosyncratic shocks, to lower survival rate of uninsured households. Then, a link to an uninsured household is more valuable than that to an insured households. Uninsured households can at least provide informal transfers in the few situations where they are not affected by a shock but their neighbors are.

## Discussion

To fight poverty, the poorest and most vulnerable households need opportunities to recover from financial losses that result from climate-related extreme events or other unexpected shocks. Microinsurance products are promoted as effective tools to address this challenge. With this study, we aimed to assess potential long-term consequences of introducing such formal insurance schemes to communities in which informal risk-sharing arrangements between smallholder farmers are prevalent. Since empirical studies have shown how diverse the transfer behavior of households can be after they have purchased insurance [19, 21–26], it is important to explicitly take transfer decisions into account when assessing the effectiveness of the combination of these risk-coping instruments. To systematically distinguish situations where formal insurance complements existing risk-sharing arrangements from situations in which harmful consequences on the resilience of smallholders emerge, we developed an agent-based model with formal and informal insurance options and combined this with social network analyses. We explicitly included two behavioral implications of the availability of insurance on informal transfers: We assumed that insured households in the social network either continue to engage in informal risk-sharing arrangements or refuse transfers after taking up formal insurance.

Our model results showed that the introduction of formal insurance can have serious consequences, even if insured households maintain private transfers. Informal risk-sharing is only effective with a sufficient number of strong actors. Insured households that do not contribute at all or are, due to premium payments, only able to contribute a small amount, reduce the strength of informal transfers. Similarly, in the case of covariate shocks where many households are affected simultaneously, purely informal risk-coping cannot be considered effective. In this case, the solidarity of insured households contributes to saving some uninsured households, which makes formal insurance a valuable complement to informal private transfers. As extreme weather events like droughts or floods which cause such types of shocks are expected to occur more frequently under climate change [12–15], formal insurance will become increasingly important in the future. In any case, the resilience of the financial resources to shocks is the highest for insured households. In general, households can financially benefit from not investing in any form of risk protection, but this is at the expense of a small number of uninsured households which can survive and is therefore the riskiest option. Taking part in informal risk-coping within social networks lowers this risk and, at the same time, still leads to individual budgets which are on average higher than those of insured households. From this perspective, it is understandable that insured households might stop their contribution to uninsured households.

The use of an agent-based model in this theoretical study enabled a systematic analysis of external conditions as well as human behavior in a social network, and allowed to disentangle effects of formal and informal insurance on the resilience of smallholders to shocks. With the particular focus on the role of monetary transfers as a risk-coping mechanism emerging from the social network and insurance as an external factor, we provided a differentiated view on drivers contributing to vulnerability and resilience in coupled human-environmental systems [49]. Here, the combination of agent-based modelling with social networks was crucial [50]. It allowed, on the one hand, to integrate individual behavior explicitly and distinguish responses to the introduction of formal insurance depending on the insurance status of the households. On the other hand, it was possible to assess how these specific decisions affect other households given the limited the range of interaction with few neighboring households defined by the imposed network structure. Both aspects, household behavior and network characteristics, could be modified and tested separately which contributed to an improved mechanistic understanding that would not have been possible without the use of these two methods.

Still, the model results should be seen in light of our rather stylized conceptualization that entails some limitations which narrow the external validity of our conclusions to some degree. Specifically, the assumption that all households have the same initial budget, income level and annual living costs, the actuarially fair insurance design with losses being covered completely and the stylized network offer plenty of potential for further studies.

In particular, if households had different financial resources, the decision to insure and potential changes in transfer behavior could be included into the model in more detail. When limited economic means alone inhibit some households from purchasing insurance [4, 42], uninsured households would on average have lower assets at their disposal which might increase their dependence on informal support. Given the different characteristics of the shock events this could have various consequences. On the one hand, the capacity of insured households to support their peers might be substantially lowered through premium payments even though they have more financial resources available than their uninsured peers. As a result, the negative effects of introducing formal insurance might potentially be even greater than that revealed in our analyses, as poor uninsured households could be less often able to sustain themselves. On the other hand, wealthy households with insurance might still be able to make effective contributions to informal risk-sharing when the premium payments only cover a small share of their available budget. In this case, informal risk-sharing could probably increase the overall welfare and formal insurance might pay-off not only for the insured households themselves but also for their uninsured peers.

Furthermore, when it is assumed that not all households with the possibility to insure are willing to do so, a heterogeneous income distribution would allow to include changes in transfer behavior more specifically. In addition to the two extreme cases of full support, on the one hand, and complete decline of solidarity by insured households, on the other hand, as shown in our analyses, a more nuanced sharing scenario could be taken into account. One could, for example, assume that insured households help only those who cannot afford insurance but do not support households with sufficient financial resources who have deliberately chosen not to purchase insurance [22]. In this case, the poorest might benefit from their insured peers being protected against income shocks and richer households that do not get any help from others might be able to cover losses from shocks through their own budget, making the lack of informal support potentially less severe.

Additional dynamics could also be observed when taking into account a discrepancy between actual losses and insurance payouts. This could be due to basis risk, i.e. when index insurance measurements do not match the suffered damages, or due to a contract with reduced insurance coverage that requires insured households to pay for parts of their losses. Similarly, when insurance is actuarially unfair, i.e. when it comprises an insurance load that covers administrative costs, moral hazard and adverse selection or allows the insurance company to make profit, households get on average less return in case of a loss compared to what they have paid as premium [51, 52]. In these cases, informal support might get more important also for insured households that could profit from neighbors taking over losses not covered by insurance [7, 25, 26, 29].

Another step towards increased realism would be the use of empirical social networks in which households differ in their number of neighbors. Heterogeneity with respect to the network position could affect the resilience to shocks of uninsured households. If a household in need has few neighbors and is not connected to those having enough resources to share, the informal support might not be able to effectively cover its losses. Next to further alignment with context-specific details, the model could also be used to test other behavioral theories and try to replicate empirically observed practices [53]. This could include explicit assumptions on risk-sharing motives such as tit-for tat [54] or indirect reciprocity [55, 56] combined with punishment to those who free-ride on the cooperation of others [57]. On the other hand, the model could be extended to account for rather unexpected behavior, such as insured households increasing their informal transfers driven by guilt-aversion [58] or uninsured households sanctioning their insured peers for their privilege through reducing contributions to a public good [59]. The latter could, for example, be analyzed by explicitly modelling a common property grazing system [60]. Incorporating these diverse aspects into the model would further allow for increased interaction between empirical and model-based studies, as results obtained from different model assumptions can also inspire additional empirical research [61].

Disentangling cause-effect relationships of empirically observed patterns of transfer behavior and exploring their long-term implications is valuable for sustainable insurance design. From our model results, we can derive that insurance products should be developed in close alignment with existing risk-coping arrangements in order to maintain these crucial structures and use their benefits effectively. If an extensive uptake of formal insurance results in crowding-out of informal networks, this bears consequences not only for households that cannot afford formal insurance. Social networks include adaptive strategies going beyond financial support such as information sharing, access to resources or equipment, or conflict intervention [62]. Moreover, embeddedness within communities promotes forward-looking decisions that can contribute to finding ways out of impoverishment [63]. Offering insurance to groups rather than individuals or families could be a suitable approach to harness formal insurance but at the same time maintain informal relationships [18, 64–66]. The network would in this case pay the insurance as a whole which allows internal agreements on contributions to the premium. Thus, every household could provide a fair share to a formal contract that protects the whole group. Existing informal associations have been successfully addressed in the context of savings [67] and microfinance [68]. Given different group structures and power relations, group insurance is, however, not equally well applicable to all informal networks [66]. Furthermore, as underlined by our simulation model, idiosyncratic risks can be covered at least partially by informal risk-sharing and only when facing covariate risks households are highly dependent on formal protection. Taking this risk layering into account by including informal risk management in the design of formal insurance products could reduce insurance costs, which would allow more households to participate and decrease social inequality [69–71].

## Conclusion

Introducing formal insurance in communities with functioning informal risk-sharing arrangements can have a crucial impact on household welfare, especially for those who do not have access to formal insurance. Our simulation results show that when insured households become unwilling to help households without insurance and withdraw their contribution to informal transfers, this largely reduces the ability of households without access to insurance to cope with income losses. Uninsured households alone cannot provide the assistance that is required by households in need. We also observe that even if the solidarity of insured households remains unchanged, uninsured households may be worse off than without some of their neighbors being insured. This is because the regular premium payments that insured households have to make reduce their ability to contribute to informal transfers. However, in the case of shocks that affect many households at the same time (such as droughts or floods), formal insurance complements informal risk sharing, since in this case uninsured households cannot do much to help their peers.

Overall, our study offers new perspectives on the interplay of formal and informal risk-coping instruments that complement existing empirical research. The combination of agent-based modelling and social networks made it possible to systematically analyze the effects of external conditions as well as human interaction on the resilience of smallholders to shocks. By embedding a broad range of theoretical and experimental findings, our results allow conclusions on potential unintended consequences that the introduction of formal insurance may have on the functioning of informal transfers in a long-term perspective. These potential feedbacks have to be kept in mind for an effective design of insurance policies as only if formal insurance and existing risk-sharing mechanisms are well aligned, they provide a good basis for achieving the goal of eradicating poverty worldwide in a sustainable manner. However, since our results are based on a theoretical simulation model, which by its nature involves a number of simplifying assumptions, the specific empirical circumstances must be taken into account in any case when evaluating an appropriate insurance design. To this end, our analyses provide an orientation on which potential side effects should be borne in mind.

## Supporting information

S1 Appendix Model documentation. Description of the main processes of the simulation model in a structured form based on the ODD+D protocol.
(PDF)

S2 Appendix Parameter selection. Description of the selection of the parameter combinations of living costs, shock probability and shock intensity.
(PDF)

S3 Appendix Additional results for idiosyncratic shocks (selected parameter combination). Additional results for idiosyncratic shocks for the selected parameter combination used in the main analysis on networks with different number of neighbors and rewiring probability.
(PDF)

S4 Appendix Additional results for idiosyncratic shocks (all parameter combinations). Additional results for idiosyncratic shocks for all economically feasible parameter with different levels of consumption and network characteristics (number of neighbors, rewiring probability).
(PDF)

S5 Appendix Additional results for covariate shocks. Additional results for covariate shocks with different levels of consumption and shock extension.
(PDF)
