# Supplementary material for: Informal risk-sharing between smallholders may be threatened by formal insurance: Lessons from a stylized agent-based model
Source: PLoS One. 2021 Mar 19;16(3):e0248757. doi: 10.1371/journal.pone.0248757 (PMC7978336; doi:10.1371/journal.pone.0248757)
Supplement: S1 Appendix — Description of the main processes of the simulation model in a structured form based on the ODD+D protocol. (PDF) [file pone.0248757.s001.pdf]

Model documentation

In the following, we describe the main processes of the simulation model in a structured form based on the ODD+D protocol (Müller et al., 2013). A conceptual diagram of the model entities and their relationships is shown in Fig S1.

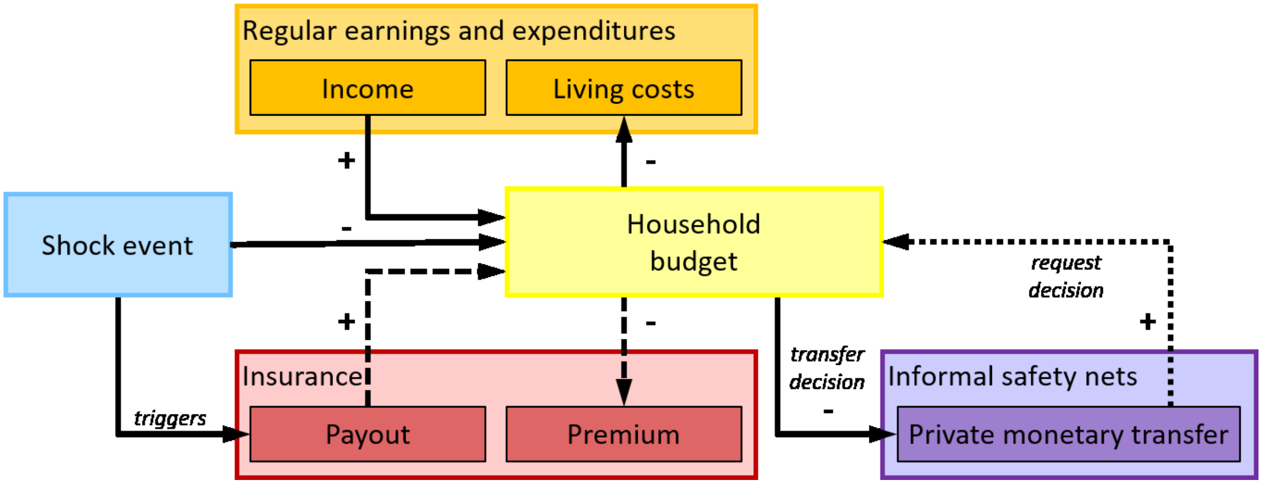

Fig S1. Conceptual diagram of the model showing the household budget, its main drivers and their relationships. Long-dashed relations are only important for insured households, short-dashed relations only for uninsured households.

Outline		Guiding questions	Description
I) Overview	I.i Purpose	I.i.a What is the purpose of the study?	The purpose of the study is to assess the impact of microinsurance and informal safety nets on the resilience of smallholders. We systematically compare the effectiveness of formal insurance and informal risk-sharing to buffer income shocks given different economic needs and characteristics of extreme events. We explicitly distinguish two types of behavior of insured households with regard to private monetary transfers.
		I.i.b For whom is the model designed?	Due to the stylized character of the model, it is primarily designed for the scientific community to understand impacts of the combination of formal and informal insurance. However, with adaptation to specific regions, it could be also valuable to increase understanding of political decision-makers and insurance providers.
	I.ii Entities, state variables, and scales	I.ii.a What kinds of entities are in the model?	There is a single type of agents representing smallholder households. Each household is linked to other households in an undirected small-world network (Watts and Strogatz, 1998) with given number of neighbors and rewiring probability.
		I.ii.b By what attributes (i.e. state variables and parameters) are these entities characterized?	- **budget:** current budget of a household determined by its initial budget, regular earnings, regular expenses, budget loss due to shocks, insurance premium payment, insurance payout in case of a shock and private monetary transfers to or from other households<br>- **insurance:** status of a household whether insured or not<br>- **shock affection:** status of a household whether affected income shocks or not<br>- **donation willingness:** status of household whether willing to transfer or not (see III.iv.a for details)<br>- **transfer-behavior:** type of behavior that the household follows when asked for transfers (see III.iv.a for details)<br>- **links:** households are connected to other households via undirected links
		I.ii.c What are the exogenous factors / drivers of the model?	Households are exposed to income shocks whose occurrence is determined stochastically.
		I.ii.d If applicable, how is space included in the model?	Space is not explicitly included in the model. However, the small-world network algorithm allows to create networks with varying levels of heterogeneity which can be seen as roughly representing different spatial clustering in villages. Low rewiring probabilities lead to highly clustered regular networks whereas high rewiring probabilities create poorly clustered random networks.
		I.ii.e What are the temporal and spatial resolutions and extents of the model?	The model uses discrete time steps. One time step (tick) represents one year. The time horizon of the model is 50 years. Space is not explicitly included.

	I.iii Process overview and scheduling	I.iii.a What entity does what, and in what order?	- **Initialization:** set up of households (initial budget, insurance status, donation willingness) and small-world network<br>- In every tick:<br>• All households (synchronous):<br>▪ Budget increases by **income** and decreases by **annual living costs**<br>▪ Insured households: pay insurance **premium**<br>▪ Shock affected households: budget decreases by **shock intensity**<br>▪ Insured households affected by shock: receive **payout**<br>• All households (random order):<br>▪ Households in need **request transfers** from randomly chosen households they are connected to in the network<br>▪ Requested households **transfer money** to requesting households according to transfer behavior<br>• **Check for surviving households:** If household's budget is below zero, household has to leave the system.
II) Design Concepts	II.i Theoretical and Empirical Background	II.i.a Which general concepts, theories or hypotheses are underlying the model's design at the system level or at the level(s) of the submodel(s) (apart from the decision model)? What is the link to complexity and the purpose of the model?	- We assume that households have access to formal insurance and traditional informal safety nets to secure themselves against income shocks. These shocks can be idiosyncratic shocks, hitting the households independently (such as health shocks), or covariate shocks, affecting many households at the same time (such as drought shocks).<br>- Complexity results from the feedback between the dynamics of the budget of individual households and monetary transfers between households in networks.<br>- By explicitly including two types of behavior of insured households with regard to private monetary transfers, the model contributes to the debate of unintended side effects of formal insurance schemes and helps to identify long-term effects and structural peculiarities that influence the outcome.
		II.i.b On what assumptions is/are the agents' decision model(s) based?	The decision models for transfer provision are based on observations from case studies and reflect behavior with and without solidarity of insured households.
		II.i.c Why is a/are certain decision model(s) chosen?	Empirical observations show mixed results with respect to the transfer behavior of insured households. Therefore, we have chosen two strategies of transfer decisions which reflect behavior with and without solidarity towards uninsured households. In one simulation run, all households decide on their transfers according to the same strategy. For the first strategy, all households show solidarity, i.e. they transfer whenever they can afford it. In a second strategy, we assume that only uninsured households show solidarity and contribute to informal risk-sharing whenever they can afford it; insured households do not

			transfer at all. We have implemented the two decision rules to compare the effects of both behaviors on the resilience of smallholders.
		II.i.d If the model / a submodel (e.g. the decision model) is based on empirical data, where does the data come from?	Most parts of the model are not directly based on empirical data. The values of household characteristics are chosen in a range derived from literature on microinsurance and informal transfer networks in different countries (for specific references see III.iv.b). Furthermore, the combined parameter space for income, living costs, shock probability and shock intensity is reduced based on economic constraints (for details see III.iv.c).
		II.i.e At which level of aggregation were the data available?	Not applicable.
	II.ii Individual Decision Making	II.ii.a What are the subjects and objects of decision-making? On which level of aggregation is decision-making modeled? Are multiple levels of decision making included?	There is one **level of decision making**, the household level. Households are the **subject** of decision making. The monetary transfer provision from wealthy households to households in need in the network is the **object**.
		II.ii.b What is the basic rationality behind agents' decision-making in the model? Do agents pursue an explicit objective or have other success criteria?	- **Transfer request:** Each household's objective is to maintain prosperity with a budget above or equal to zero. Households with a budget below zero request help from other agents with a budget above zero in their network.<br>- **Transfer provision:**<br>• **Solidarity:** Households transfer whenever they can afford it (i.e. have a budget above zero). This implies that households may assume that the requesting household will return the transfer in the future if they need support themselves. Since, in the simulated scenarios, insurance covers all losses, this will only occur for uninsured households.<br>• **No solidarity:** Only uninsured households show solidarity and contribute to informal risk-sharing whenever they can afford it (i.e. have a budget above zero); insured households do not transfer at all. This implicitly includes that they are (1) not dependent on reciprocal behavior of other households because shocks are fully covered by the insurance and (2) not willing to transfer as they have more costs due to the insurance that uninsured households avoided.
		II.ii.c How do agents make their decisions?	- Agents' decision rules are implemented as if-then rules.

			- **Transfer request:** Households in need randomly pick one of the households in their network with budget above zero. If the request cannot be fulfilled by one single agent, households continue requesting the missing amount from other agents in their network.<br>- **Transfer provision:** Households that have been requested for a transfer decide how much to transfer based on one of two decision rules:<br>• **Solidarity:** The transfer amount is determined by the request and their own budget. The minimum budget of a donating household after the transfer is zero.<br>• **No solidarity:** Insured households do not transfer at all; uninsured households show solidarity. In this case, the transfer amount is determined according to the same rules as for solidarity.
		II.ii.d Do the agents adapt their behavior to changing endogenous and exogenous state variables? And if yes, how?	Yes. Households adapt the transfer amount to the requested amount and their own budget. It is incorporated that donors do not put themselves at financial risk through transfers. Therefore, the minimum budget of a donor after a transfer is zero. On the other hand, the household in need should not get too rich through the help of others. The maximum budget that can be achieved through transfers is thus also zero.
		II.ii.e Do social norms or cultural values play a role in the decision-making process?	Transfer behavior with solidarity is implicitly based on expected reciprocity.
		II.ii.f Do spatial aspects play a role in the decision process?	No, space is not explicitly included in the model.
		II.ii.g Do temporal aspects play a role in the decision process?	Households make decisions based only on the current state of the system.
		II.ii.h To which extent and how is uncertainty included in the agents' decision rules?	Uncertainty is not included in the decision making.
	II.iii Learning	II.iii.a Is individual learning included in the decision	No, learning is not included.

		process? How do individuals change their decision rules over time as consequence of their experience?	
		II.iii.b Is collective learning implemented in the model?	No.
	II.iv Individual Sensing	II.iv.a What endogenous and exogenous state variables are individuals assumed to sense and consider in their decisions? Is the sensing process erroneous?	Households adapt their decision making to variables of households they are linked to in the network (see II.iv.b).
		II.iv.b What state variables of which other individuals can an individual perceive? Is the sensing process erroneous?	Requested households sense the amount asked for by the household in need. The sensing is not erroneous, i.e. the households always perceive the true requested amount. Households in need do not know the insurance status of their neighbors.
		II.iv.c What is the spatial scale of sensing?	Not applicable directly as space is not explicitly included in the model. Concerning sensing in the network, households include their direct neighbors in the network only.
		II.iv.d Are the mechanisms by which agents obtain information modeled explicitly, or are individuals simply assumed to	Agents are assumed to know the values of the sensed variables.

		know these variables?	
		II.iv.e Are costs for cognition and costs for gathering information inclu-ded in the model?	No.
	II.v Individual Prediction	II.v.a Which data uses the agent to predict future conditions?	Households do not predict future conditions.
		II.v.b What internal models are agents assumed to use to estimate future conditions or consequences of their decisions?	Not applicable.
		II.v.c Might agents be erroneous in the prediction process, and how is it implemented?	Not applicable.
	II.vi Interaction	II.vi.a Are interactions among agents and entities assumed as direct or indirect?	Interactions between households are direct. Households in need request money from households they are linked to in the network which then decide how much to transfer.
		II.vi.b On what do the interactions depend?	Interactions depend on the budget of the household in need and the requested household as well as the transfer decision and insurance status of the requested household.
		II.vi.c If the interactions involve communication, how are such communications represented?	Communication is represented by transfer request and provision. The transferred amount is reduced from the budget of the giving household and added to the budget of the household in need.

		II.vi.d If a coordination network exists, how does it affect the agent behavior? Is the structure of the network imposed or emergent?	- The network does not directly influence the behavior, but requests for transfers are only possible between directly linked households.<br>- The network structure is imposed during the initialization of the model and is kept constant (i.e. static) for a simulation run.
	II.vii Collectives	II.vii.a Do the individuals form or belong to aggregations that affect, and are affected by, the individuals? Are these aggregations imposed by the modeler or do they emerge during the simulation?	Households are connected in a network that influences their interaction range for monetary transfers. The network is imposed during the initialization of the model and is kept constant (i.e. static) during the simulation run. The network is based on a stylized small-world network.
		II.vii.b How are collectives represented?	Collectives are represented as a network.
	II.viii Heterogeneity	II.viii.a Are the agents heterogeneous? If yes, which state variables and/or processes differ between the agents?	All agents have the same set of state variables and processes. A fixed proportion of the households is insured, the rest is uninsured. The population is homogeneous with all households having the same initial budget, income level and annual living costs.
		II.viii.b Are the agents heterogeneous in their decision-making? If yes, which decision models or decision objects differ	Households take the same decisions on whom to ask for transfers and how much to transfer. However, based on their insurance status, households' choices on transfer provision can be heterogeneous (see II.ii.b or III.iv.a).

		between the agents?	
	II.ix Stochasticity	II.ix.a What processes (including initialization) are modeled by assuming they are random or partly random?	- Insurance status is assigned randomly.<br>- Income shocks occur randomly (different for idiosyncratic and covariate shocks, see III.iv.a).<br>- Households in need request transfers from households randomly chosen among the households they are linked to in the network.
	II.x Observation	II.x.a What data are collected from the ABM for testing, understanding, and analyzing it, and how and when are they collected?	For parameter variations conducted with the R-package nlrx (Salecker et al., 2019), we collect for every time step the states of (NetLogo variable names are given in brackets):<br>- **Resilience:** Fraction of surviving households (*fraction-active*) and surviving uninsured households (*fraction-active-uninsured*)<br>- **Budget:** Total budget of all (*total-budget*), insured (*total-budget-insured*) and uninsured households (*total-budget-uninsured*) and mean budget of all (*mean-budget*), insured (*mean-budget-insured*) and uninsured households (*mean-budget-uninsured*)<br>- **Transfer requests:** Number of households that need help per time step (*requesting-households*), the amount of money they need per time step (*total-money-needed*) and the total amount of money needed up to that time step (*cum-money-needed*)<br>- **Transfer provision:** Total transfer given up to that time step by all (*total-transfer*), active (*total-transfer-active*), insured (*total-transfer-given-insured*), uninsured (*total-transfer-given-uninsured*) and uninsured active households (*total-transfer-given-uninsured-active*) and transfer received by uninsured active households (*total-transfer-received-uninsured-active*)<br>- **Inequality:** GINI coefficient of all (*get-gini*), insured (*get-gini-insured*) and uninsured households (*get-gini-uninsured*)<br><br>For each household, we collect for every time step:<br>- **Budget:** The total budget of a household (*budget*) and if a households' budget is above or equal to zero (*active*)<br>- **Transfer:** If a household is willing to provide transfers (*donation*-willingness), the total amount of money received by (*received*) and transferred to (*given*) other households, the total number of transfers (*total-donates*) and transfers per time step (*current-donates*) and the total number of requests (*total-requests*) and requests per time step (*current-requests*)<br>- **Shock:** Whether a household is affected by a shock in that time step (*shock-affected*) and how often a household was affected by a shock (*shock-affected-sum*)

			For each link, we collect for every time step:<br>- **Transfer:** The total amount of money (*total-flow*) and the amount per time step (*current-flow*) transferred between the two households in the direction of the link and the number of transfers on that link (*number-flows*)<br>- **Resilience:** If a link is active, i.e. if both connected households have a budget above zero (*active-link*)<br><br>In the graphical user interface, we plot the values of the following variables for each time step:<br>- **Resilience:** Fraction of surviving households (*fraction-active*) and surviving uninsured households (*fraction-active-uninsured*)<br>- **Budget:** Mean budget of all (*mean-budget*), insured (*mean-budget-insured*) and uninsured households (*mean-budget-uninsured*)<br>- **Transfer provision:** Current transfer per time step given by all, insured and uninsured households<br>- **Inequality:** GINI coefficient of all (*get-gini*), insured (*get-gini-insured*) and uninsured households (*get-gini-uninsured*)
		II.x.b What key results, outputs or characteristics of the model are emerging from the individuals? (Emergence)	We can observe the effectiveness of formal and informal insurance given different economic needs (income, living costs), characteristics of extreme events (shock probability, shock intensity, type of shock), transfer behavior (solidarity, no solidarity) and network properties (average degree, rewiring probability) on the resilience of the households, i.e. the fraction of surviving households, and their budget.
III) Details	III.i Implementation Details	III.i.a How has the model been implemented?	The model has been implemented in NetLogo 6.1.1.
		III.i.b Is the model accessible and if so where?	The model is available at CoMSES Net: https://www.comses.net/codebases/0f779ef3-5c89-4fe0-be7f-2757f9789028/releases/1.0.1/
	III.ii Initialization	III.ii.a What is the initial state of the model world, i.e. at time t=0 of a simulation run?	At the beginning of each simulation, households are initialized with initial budget and insurance status. Shock type and households' transfer behavior is defined according to the chosen scenarios (see III.iv).
		III.ii.b Is initialization always the same, or is it allowed to vary among simulations?	Initialization varies between different scenarios (for details of the implementation of the scenarios see III.iv.a).

		III.ii.c Are the initial values chosen arbitrarily or based on data?	Initial values are arbitrarily chosen.
	III.iii Input Data	III.iii.a Does the model use input from external sources such as data files or other models to represent processes that change over time?	The model does not use input data to represent time-varying processes.
	III.iv Submodels	III.iv.a What, in detail, are the submodels that represent the processes listed in ‘Process overview and scheduling’?	<u>**Setup processes**</u><br>*Function name: setup*<br><br>**Household setup**<br>*Function name: setup-households*<br>N_{H} households are created and initialized with an initial budget Y^0. Initial budget and income level is the same for all households. A shock series is determined for the simulated time span T. The calculation of the shock series is different for idiosyncratic shocks hitting the households independently and covariate shocks affecting many households at the same time:<br>- **Idiosyncratic shocks:** For each household, the shock series is determined individually. Shocks occur with probability p_{s}.<br>- **Covariate shocks:** A shock series is determined for the whole village. Shocks occur with probability $p_{\mathrm{V}} = p_{\mathrm{s}}/p_{\mathrm{H}}$. In time steps where the village is affected by a shock, individual households are affected with probability p_{H}. This results in an overall shock probability $p_{\mathrm{s}} = p_{\mathrm{V}} \times p_{\mathrm{H}}$ for an individual household. We distinguish between cases in which all households without exception are affected by the shock ($p_{\mathrm{H}} = 1$) and cases in which some households are exempted ($p_{\mathrm{H}} = 0.8$), for example by a more favorable geographical location in case of floods or an agricultural management strategy more adapted to drought risks.<br>To make the strategies comparable, in one repetition the shock series of one specific household is the same for every risk-coping instrument.<br><br>**Network setup**<br>*Function name: create-small-world-network*<br>A small-world network is generated using the *generate-watts-strogatz* primitive in the NetLogo Nw Extension which is based on the Watts-Strogatz model (Watts and Strogatz, 1998). Essentially, the algorithm creates a ring of households with each node connected to N_{N} nodes on either side. Each link is rewired with rewiring probability p_{r}. To allow for the control of the transfers in both directions of a link

separately, the algorithm is slightly modified so that directed links to $N_{\mathrm{N}}/2$ households are created on one side of the agent. After rewiring, a link in the opposite direction is established for each existing link. This leads to an undirected small-world network with average degree N_{N}. Based on data from Ethiopia, a household is on average willing to transfer to 3.8 households (Takahashi et al., 2018). Therefore, we have chosen an average neighborhood size of $N_{\mathrm{N}} = 4$. To consider the effects of more or less neighbors, we additionally present the results for $N_{\mathrm{N}} = 8$ and $N_{\mathrm{N}} = 2$. We compare two types of spatial clustering with low ($p_{\mathrm{r}} = 0.2$) and high ($p_{\mathrm{r}} = 0.8$) rewiring probability.

Insurance targeting

Function name: insurance-take-up

An insurance take-up rate γ is given. Among all households $\gamma \times N_{\mathrm{H}}$ (rounded down if necessary) are randomly selected to be insured. Insured households insure their complete income.

Donation willingness

Function name: set-donation-willingness

If transfers between households are considered, households' willingness to provide transfers is set to 1 for uninsured households and insured households showing solidarity and 0 for insured household not showing solidarity. For the reference case where no transfers are considered, households' willingness to provide transfers is set to 0 for all households.

Processes in every time step

Function name: go

Every time step is divided into two phases. In the first phase, households execute processes without interaction in the network. The processes run sequentially and in the following order: regular earning, regular expenses, insurance premium payment, budget loss due to shocks, and insurance payout. In the second phase, after all households have completed the first one, households are selected in random order to execute transfer requests if necessary. Since the insurance covers all losses, only uninsured households may get into the situation of having to request transfers from the neighbors with whom they have social ties. Budgets of households in need and households providing transfers are updated after each transfer according to the amount received and provided. At the end of each time step, households whose budget is less than zero have to leave the system.

Phase I:

Regular earnings

Function name: annual-income

Households add a fixed amount I to their budget as annual income.

Regular expenses
Function name: annual-consumption
Households consume a fixed amount C of their budget to cover their annual living costs.

Budget loss due to shocks
Function name: shock-loss
Shocks occur with intensity S. If according to its individual shock series a household is affected by a shock, the budget of that household is reduced by this amount.

Insurance premium and payout
Insured households insure their complete income.

Payout
Function name: insurance-payout
The insurance covers the actual losses a household suffers from. The payout α in case of a shock is $\alpha = S$.

Premium
Function name: insurance-premium
The insurance is actuarially fair. Insured households have to pay a yearly premium β equal to the expected payout: $\beta = p_s \times S$.

Phase II: Informal monetary transfers
Function name: informal-transfers

Transfer request
Function name: transfer-request
Households request monetary transfers from households they are linked to in the network if their budget is below zero. A requesting household i requests a transfer amount $T_{i,\mathrm{req}}$ that covers its debts Y_i: $T_{i,\mathrm{req}} = |Y_i|$. A household in need can ask households in its network for help which have a budget above zero. The household randomly picks one of the possible households. The budgets are updated after every transfer. Households continue to ask until they obtain the requested amount or until no more households are able to support them.

Transfer provision

Function name: transfer-money, transfer-amount

Households are potential donors if their budget Y_j is above zero. Depending on the scenario, all households show solidarity or only uninsured households show solidarity and insured households do not transfer. Households in need do not know the insurance status of their neighbors.

- **Solidarity:** All potential donors are willing to transfer if requested. If the requested amount is smaller than their own budget, the amount transferred T_{ij} equals the requested amount $T_{i,\mathrm{req}}$, otherwise they transfer their complete budget Y_j: $T_{ij} = \min\{Y_j; T_{i,\mathrm{req}}\}$
- **No solidarity:** Potential donors that are uninsured behave as in the solidarity case. Insured households do not transfer.

Household budget equation

All processes sum up to the following equation for the budget $Y_i(t)$ of household i at time step t:

$$Y_i(t) = \begin{cases} Y_i(t-1) + I - C - \beta - S_i + \alpha_i + \sum_{j \in N_i} T_{ij}(t) & \text{for insured households} \\ Y_i(t-1) + I - C - S_i + \sum_{j \in N_i} T_{ij}(t) & \text{for uninsured households} \end{cases}$$

with income I, annual living costs C and premium β. The shock intensity S_i equals S if a household is affected by a shock and is zero otherwise. The same holds true for the insurance payout α_i. For $t = 1$ the budget of the previous time step $t - 1$ is given by the initial budget Y^0.

N_i denotes all households that share a link with household i and $T_{ij}(t)$ is the transfer between households i and j at time step t. Transfers can be positive, negative or zero for uninsured households (receiving and providing transfers) and negative or zero for insured households (only providing transfers).

Check for surviving households

If a household's budget is below zero at the end of a time step, the household has to leave the system.

III.iv.b What are the model parameters, their dimensions and reference values?

Parameter	Description	NetLogo name	Unit	Standard value / range	Reference
T	Number of ticks that the model run	timesteps	Years	50	-
N_{H}	Number of households in the system	number-households	Unitless	50	-
N_{N}	Neighborhood size (small-world network)	neighborhood-size	Unitless	2, 4, 8	Takahashi et al. (2018)

			p_r	Rewiring probability (small-world network)	rewire-prob	Unitless (rate)	0.2, 0.8	-
			I	Annual household income	income-lvl	Normalized to 1	1	-
			Y^0	Initial budget	budget-init	Unitless, related to I	0	-
			γ	Insurance take-up rate	insurance-take-up-rate	Unitless (rate)	0, 0.3, 0.6	Takahashi et al. (2018)
			C	Annual living costs	consumption-lvl	Unitless, related to I	0.7 – 0.9	Matsuda et al. (2019) and Takahashi et al. (2016)
			p_s	Probability for shock occurrence	shock-prob	Unitless (rate)	0.1 – 0.3	Geng et al. (2018) and Anderberg and Morsink (2020)
			p_V	Probability for shock occurrence at village level (covariate shock)	covariate-shock-prob-vlg	Unitless (rate)	$p_\mathrm{V} = p_\mathrm{s}/p_\mathrm{H}$	-
			p_H	Probability that individual households are affected by a shock if the village is affected (covariate shock)	covariate-shock-prob-hh	Unitless (rate)	0.8, 1	-
			S	Shock intensity, i.e. budget loss due to shock	shock-intensity	Unitless, related to I	0.2 – 1	-
		III.iv.c How were submodels designed or chosen, and how were they parameterized and then tested?	The different decision submodels were chosen to build a “virtual lab” to test how transfer decisions influence overall welfare of the population and if different behavioral models lead to different outcomes. The parameter range for the network model has been adapted to literature values (see III.iv.b). The combined parameter ranges for income I, living costs C, shock probability p_s and shock intensity S need to meet two constraints: (1) Shock intensity must be high enough to make financial protection necessary and (2) formal insurance must be affordable. The resulting reduced parameter space has been adapted to economic constraints from literature values (for resulting parameter ranges see III.iv.b).					

References

Anderberg, D., Morsink, K., 2020. The introduction of formal insurance and its effect on redistribution. Journal of Economic Behavior & Organization 179, 22–45.

Geng, X., Janssens, W., Kramer, B., List, M. van der, 2018. Health insurance, a friend in need? Impacts of formal insurance and crowding out of informal insurance. World Development 111, 196–210.

Matsuda, A., Takahashi, K., Ikegami, M., 2019. Direct and indirect impact of index-based livestock insurance in Southern Ethiopia. The Geneva Papers on Risk and Insurance - Issues and Practice 44, 481–502.

Müller, B., Bohn, F., Dreßler, G., Groeneveld, J., Klassert, C., Martin, R., Schlüter, M., Schulze, J., Weise, H., Schwarz, N., 2013. Describing human decisions in agent-based models – ODD + D, an extension of the ODD protocol. Environmental Modelling & Software 48, 37–48.

Salecker, J., Sciaini, M., Meyer, K.M., Wiegand, K., 2019. The NLRX R package: A next-generation framework for reproducible NetLogo model analyses. Methods in Ecology and Evolution 10, 1854–1863.

Takahashi, K., Barrett, C.B., Ikegami, M., 2018. Does Index Insurance Crowd In or Crowd Out Informal Risk Sharing? Evidence from Rural Ethiopia. American Journal of Agricultural Economics 101.

Takahashi, K., Ikegami, M., Sheahan, M., Barrett, C.B., 2016. Experimental Evidence on the Drivers of Index-Based Livestock Insurance Demand in Southern Ethiopia. World Development 78, 324–340.

Watts, D.J., Strogatz, S.H., 1998. Collective dynamics of 'small-world' networks. Nature 393, 440–442.
