# Supplementary material for: Informal risk-sharing between smallholders may be threatened by formal insurance: Lessons from a stylized agent-based model
Source: PLoS One. 2021 Mar 19;16(3):e0248757. doi: 10.1371/journal.pone.0248757 (PMC7978336; doi:10.1371/journal.pone.0248757)
Supplement: S2 Appendix — Description of the selection of the parameter combinations of living costs, shock probability and shock intensity. (PDF) [file pone.0248757.s002.pdf]

## Parameter selection

To assess the impact of formal and informal insurance on the resilience of smallholders, the model should depict conditions in which both instruments are important and can be used effectively. This implies (1) that the shock intensity should be high enough to make financial protection necessary and (2) that formal insurance should be affordable. To select parameter combinations for living costs $C$, shock probability $p_s$ and shock intensity $S$ that fulfill these constraints, we calculate the budget change of a household per time step depending on its insurance state and the occurrence of a shock (see Table S1). Based on the probability $p_s$ with which each of these cases occur, the expected value of budget change without informal transfers equals $E[\Delta Y] = I - C - \beta$ for an insured household and $E[\Delta Y] = I - C - p_s \times S$ for an uninsured households. As the insurance is assumed to be fair ($\beta = p_s \times S$), the expected value $E[\Delta Y]$ is the same for insured and uninsured households. Based on the expected value we can select cases that are suitable for the analysis. We exclude parameter combinations with $E[\Delta Y] < 0$, as in this situations insured households lose money in every time step which would contradict the intention of insurance to protect household from monetary losses. We furthermore exclude combinations for which uninsured households do not have a negative budget change in case of a loss, i.e. we exclude cases with $E[\Delta Y] > 0$ and $I - C - S > 0$. In these cases, risk-coping instruments are not needed. Fig S1 graphically shows the resulting parameter space. For the simulation, we use values with spacing of 0.1 for the three dimensions living costs $C$, shock probability $p_s$ and shock intensity $S$.

**Table S1.** Budget change of a household per time step depending on its insurance state and the occurrence of a shock. Budget change is calculated based on the annual income $I$, living costs $C$, shock intensity $S$, premium $\beta$ and payout $\alpha$

| | Shock | No shock |
|---|---|---|
| Insured | $I - C - S + \propto - \beta$ | $I - C - \beta$ |
| Not insured | $I - C - S$ | $I - C$ |

Additionally to the mathematical restrictions, we constrain the parameters with respect to ecological and economic observations. We assume subsistence farmers that need consume a large proportion of their income to cover their living costs. Studies for livestock farmers in Ethiopia show, for example, that households consume between 69% (322 Ethiopian Birr mean monthly per capita consumption with 467 Ethiopian Birr mean monthly per capita income (Takahashi et al., 2016)) and 81% (mean annual household consumption 21,482 Ethiopian Birr with annual household income 26,631 Ethiopian Birr (Matsuda et al., 2019)) of their income. We therefore restrict the parameter for living costs to $0.7 \leq C < 1$. We furthermore assume shock probabilities in a range of $0.1 \leq p_s \leq 0.3$ which relate to empirically observed income losses. According to a study in Ethiopia, for example, the probability to lose 25-50% of the crop yield was on average 21% (Anderberg and Morsink, 2020). The rate of health shocks is similarly high. In a survey conducted in Kenya, households denoted to experience a health shock in 26.6% of the weeks in on year (Geng et al., 2018). We do not include any further restrictions for shock intensity $S$ to cover a broad range of possible outcomes. Based on suitable expected values, values for shock probabilities $p_s$ and levels of annual living costs $C$, shock intensity $S$ is within $0.2 \leq S \leq 1$. We divide all parameter ranges in equidistant steps of 0.1, which results in 52 reasonable parameter combinations that meet the constraints.

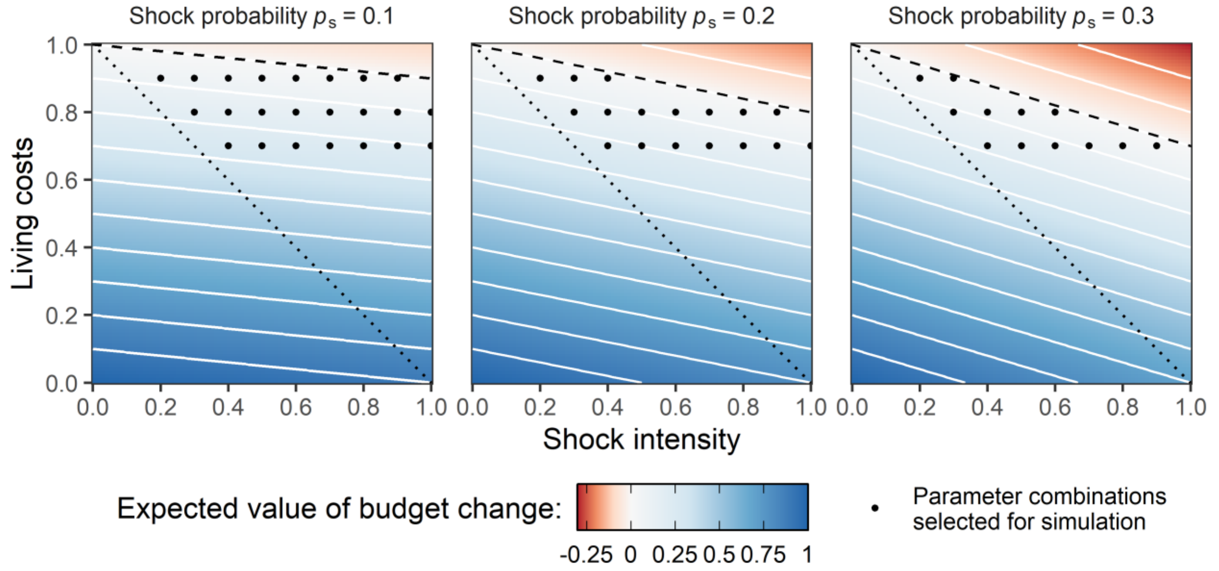


**Fig S1.** Representation of the parameter space that results from assumptions for reasonable budget changes of a household per time step. White lines connect parameter combinations with equal expected value of budget change $E[\Delta Y]$. The parameter space is divided in three main zones: For parameter combinations below the dotted line $E[\Delta Y] > 0$ and $I - C - S > 0$ applies; for parameter combinations above the dashed line $E[\Delta Y] < 0$ applies. Parameter combinations in between those two zones are suitable for the analysis. Dots represent parameter combinations that fulfill the additional ecological and economic constraints ($0.1 \leq p_s \leq 0.3, 0.7 \leq C < 1$). These parameter combinations are selected for the simulation.

## References


Anderberg, D., Morsink, K., 2020. The introduction of formal insurance and its effect on redistribution. Journal of Economic Behavior & Organization 179, 22–45.

Geng, X., Janssens, W., Kramer, B., List, M. van der, 2018. Health insurance, a friend in need? Impacts of formal insurance and crowding out of informal insurance. World Development 111, 196–210.

Matsuda, A., Takahashi, K., Ikegami, M., 2019. Direct and indirect impact of index-based livestock insurance in Southern Ethiopia. The Geneva Papers on Risk and Insurance - Issues and Practice 44, 481–502.

Takahashi, K., Ikegami, M., Sheahan, M., Barrett, C.B., 2016. Experimental Evidence on the Drivers of Index-Based Livestock Insurance Demand in Southern Ethiopia. World Development 78, 324–340.
