# Supplementary material for: Informal risk-sharing between smallholders may be threatened by formal insurance: Lessons from a stylized agent-based model
Source: PLoS One. 2021 Mar 19;16(3):e0248757. doi: 10.1371/journal.pone.0248757 (PMC7978336; doi:10.1371/journal.pone.0248757)
Supplement: S3 Appendix — Additional results for idiosyncratic shocks for the selected parameter combination used in the main analysis on networks with different number of neighbors and rewiring probability. (PDF) [file pone.0248757.s003.pdf]

Additional results for idiosyncratic shocks (selected parameter combination)

We present additional results for idiosyncratic shocks, i.e. shocks that occur for all households independently with shock probability p_s. To make the different risk-coping instruments comparable, for each household the order of shocks is determined at the beginning of each simulation run. This individual shock series is equal for the same parameter combination and random seed independent of the network characteristics and risk-coping instruments that are analyzed. We present simulation runs over 50 years for one specific parameter combination of income I, living costs C, shock probability p_{s} and shock intensity S ($I = 1, C = 0.8, p_{\mathrm{s}} = 0.3, S = 0.6$). All results show the mean over 100 repetitions. The outcomes can be compared to the observations shown in the main text where results for a small-world network with rewiring probability $p_{\mathrm{r}} = 0.2$ and an average number of four neighbors ($N_{\mathrm{N}} = 4$) are shown. Here, we analyze the fraction of surviving households (Fig S1), the fraction of surviving uninsured households (Fig S2), the total transfer (Fig S3) and the budget per surviving household (Fig S4) for (a) higher rewiring probability, (b) smaller and (c) larger average degrees.

Fraction of surviving households

We observe that rewiring probability (Fig S1A) has almost no effect on the survival rates. Only when all households show solidarity and 60% of the households are insured slightly more households survive than for a low rewiring probability. In the 60% insurance scenario, the survival rate is slightly lower when only uninsured households show solidarity. As even with higher rewiring probability, the average number of neighbors remains at $N_{\mathrm{N}} = 4$, the similar survival rates show that, in most cases, households can rely on the same number of helping households. Only if a large number of households is insured, some households benefit from a higher number of neighbors, which can result from a high rewiring probability. With a higher number of neighbors, the small contributions of insured households can more easily sum up to effective contributions. This suggests that the average number of neighbors could play an important role. We have therefore investigated which effects a smaller (Fig S1B) and larger (Fig S1C) average degree than that presented in the main text ($N_{\mathrm{N}} = 4$) has on the survival rate. As expected, a low number of neighbors ($N_{\mathrm{N}} = 2$) clearly lowers the number of households that manage to stay in the system whereas a larger number of neighbors ($N_{\mathrm{N}} = 8$) helps more households to survive. This is reasonable, since when a household in need is connected to more households, the chance that enough neighbors can and are willing to help is higher. Furthermore, the chance that even small contributions sum up to helpful transfers increases with more neighbors.

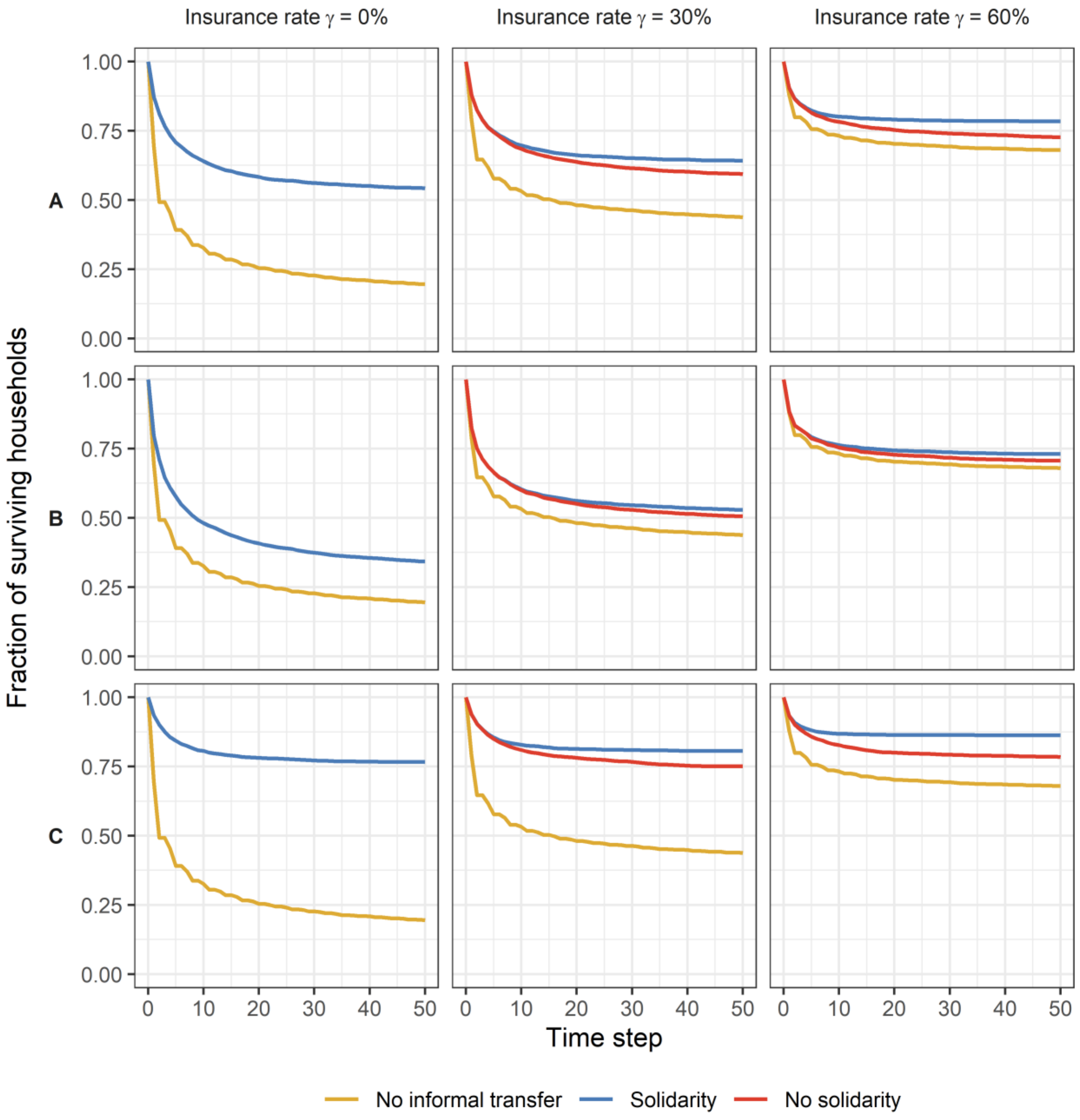

Fig S1. Fraction of surviving households for different risk-coping instruments and insurance rates ($\gamma = 0\%, \gamma = 30\%, \gamma = 60\%$) with (A) high rewiring probability ($N_{\mathrm{N}} = 4, p_{\mathrm{r}} = 0.8$), (B) small average degree ($N_{\mathrm{N}} = 2, p_{\mathrm{r}} = 0.2$) and (C) large average degree ($N_{\mathrm{N}} = 8, p_{\mathrm{r}} = 0.2$).

Fraction of surviving uninsured households

When focusing the analysis on the fraction of surviving uninsured households among the 20 households that are uninsured in every scenario, we again observe that a higher rewiring probability (Fig S2A) has only small effects. When changing the average degree (Fig S2B, Fig S2C), we observe that this influences, on the one hand, the fraction of surviving uninsured households as already observable for the fraction of surviving households and, on the other hand, the effect of solidarity from insured households. The higher the average number of neighbors, the larger is the difference between uninsured households surviving with solidarity of insured households and without. This is due to the fact that when a household in need is connected to more other households, contributions of insured households that are potentially smaller than those of uninsured households more easily sum up to effective contributions.

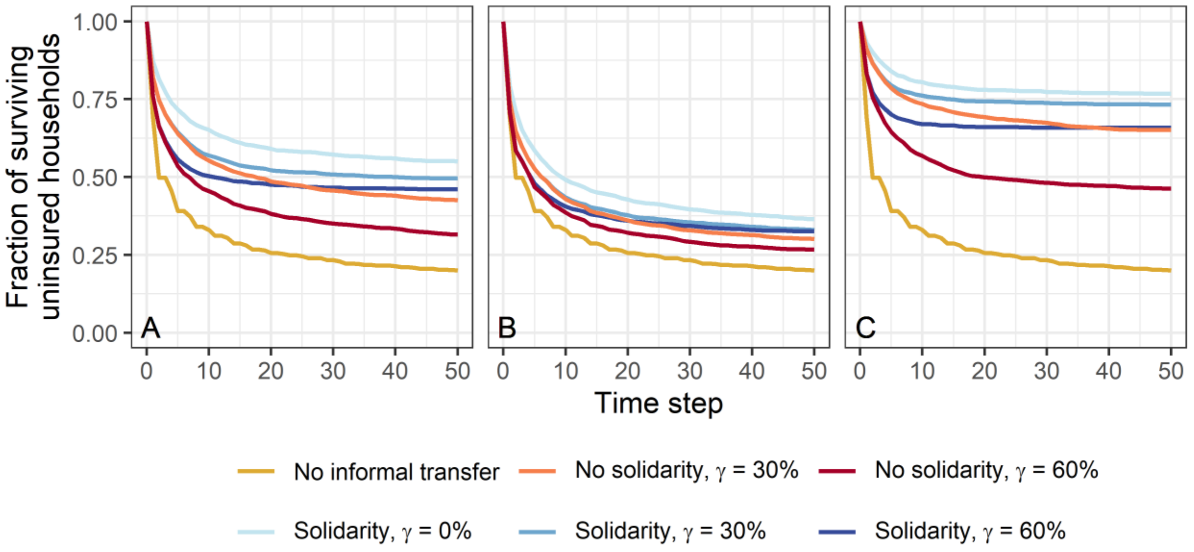

Fig S2. Fraction of surviving uninsured households among the 20 households that are uninsured in every scenario for different risk-coping instruments with insurance rates γ and (A) high rewiring probability ($N_{\mathrm{N}} = 4, p_{\mathrm{r}} = 0.8$), (B) small average degree ($N_{\mathrm{N}} = 2$, $p_{\mathrm{r}} = 0.2$) and (C) large average degree ($N_{\mathrm{N}} = 8$, $p_{\mathrm{r}} = 0.2$).

Total transfer

The total transfer received and given by the 20 households that are uninsured in every scenario per time step underlines the observations for the survival rates of uninsured households for different network conditions. The transfers in a network with high rewiring probability (Fig S3A) are, as expected from the similar survival rates, in the same range than those provided in a network with small rewiring probability. For a smaller average degree (Fig S3B) than that presented in the main text, we observe less transfers given and received by uninsured households, for a larger average degree (Fig S3C) correspondingly more. The trends that were observed for the baseline case presented in the main text hold, however, true for all additional network scenarios. The fact that households have to provide lower transfers when insured households show solidarity is even more pronounced with many neighbors. This is due to the fact that in this case the transfers tend to be spread over several shoulders and even small contributions can be effective.

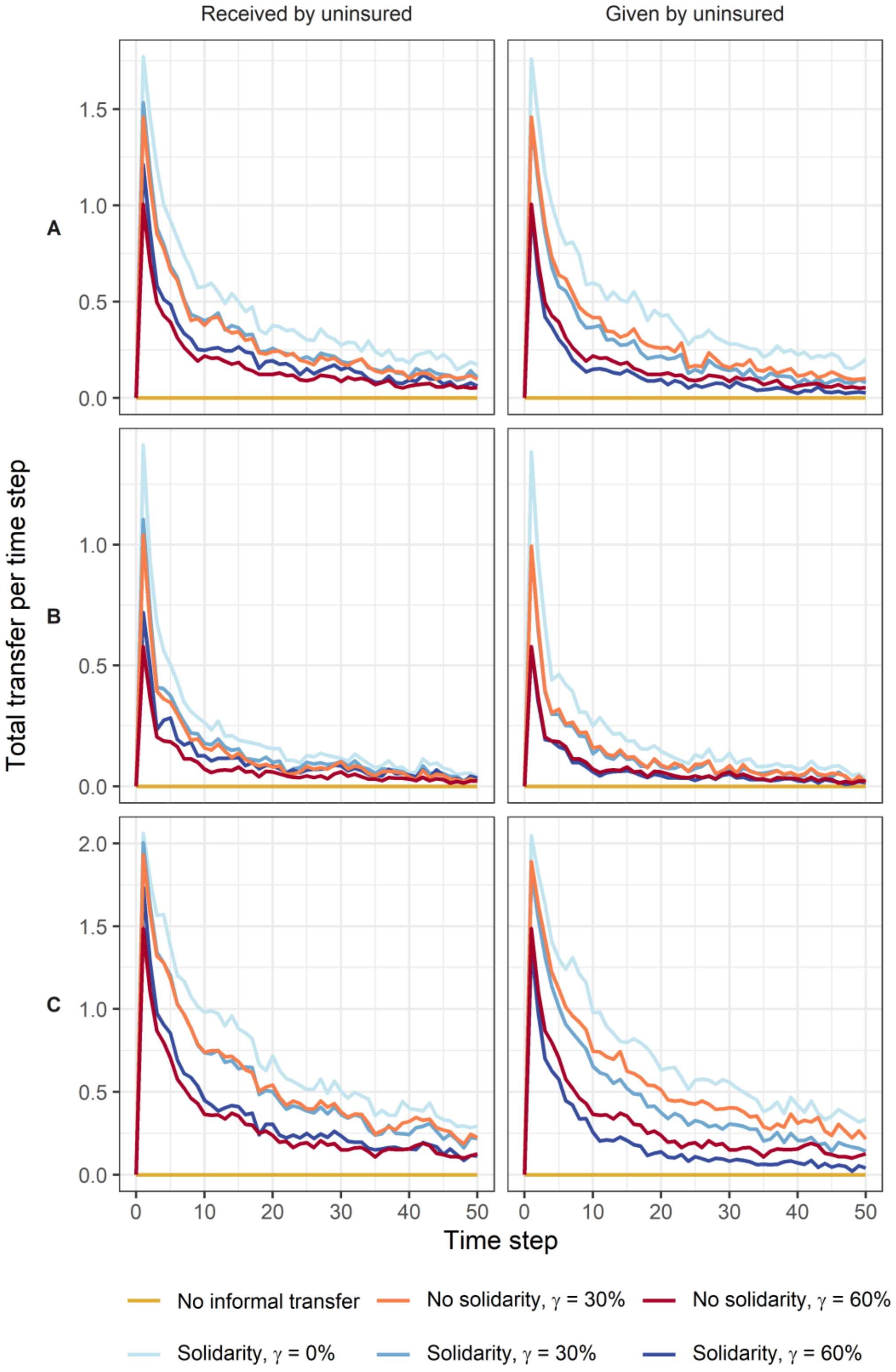

Fig S3. Total transfer received and given by all 20 households that are uninsured in every scenario per time step. Results show (A) high rewiring probability ($N_{\mathrm{N}} = 4, p_{\mathrm{r}} = 0.8$), (B) small average degree ($N_{\mathrm{N}} = 2, p_{\mathrm{r}} = 0.2$) and (C) large average degree ($N_{\mathrm{N}} = 8, p_{\mathrm{r}} = 0.2$).

Budget per surviving household

The observations for the total transfer that uninsured households receive and give for the different scenarios are directly related to the budget per surviving household. We compare the budget based on the 20 households that are uninsured in every scenario and the 15 households that are insured in every scenarios (except $\gamma = 0\%$). The straight line shows the maximum budget that an insured household can receive as reference value. As the case with no informal transfers is independent of the network characteristics, the budget per surviving household is in this case not affected by changes of rewiring probability or neighborhood size. As in all other output measures, budget as well is not affected by a larger rewiring probability (Fig S4A). However, smaller (Fig S4B) and larger (Fig S4C) neighborhood sizes have effects on the budgets of uninsured and insured households. We observe that the budget level of uninsured households increases with fewer number of neighbors. However, as for the case without informal transfers this only shows that some households suffer shocks in an order which makes it possible to accumulate a large budget. Since in this case only a few uninsured households survive, this still has an overall negative impact on resilience of uninsured households. Furthermore, we observed in the scenario presented in the main text with $N_{\mathrm{N}} = 4$ that the budget of uninsured households is only slightly affected by the solidarity of insured households. More neighbors lead to an increased budget of uninsured households in the case of solidarity compared to the case where insured households do not contribute. A larger risk-sharing group has therefore positive effects not only on the survival rate of uninsured households but also helps them to secure their financial resources. However, as this effect is largely dominated by the contributions of insured households this comes at the cost of lower budget in this subgroup. As transfers are not paid back, insured households will end up with budgets far lower than that of uninsured households which might affect their willingness to contribute to informal risk-sharing arrangements.

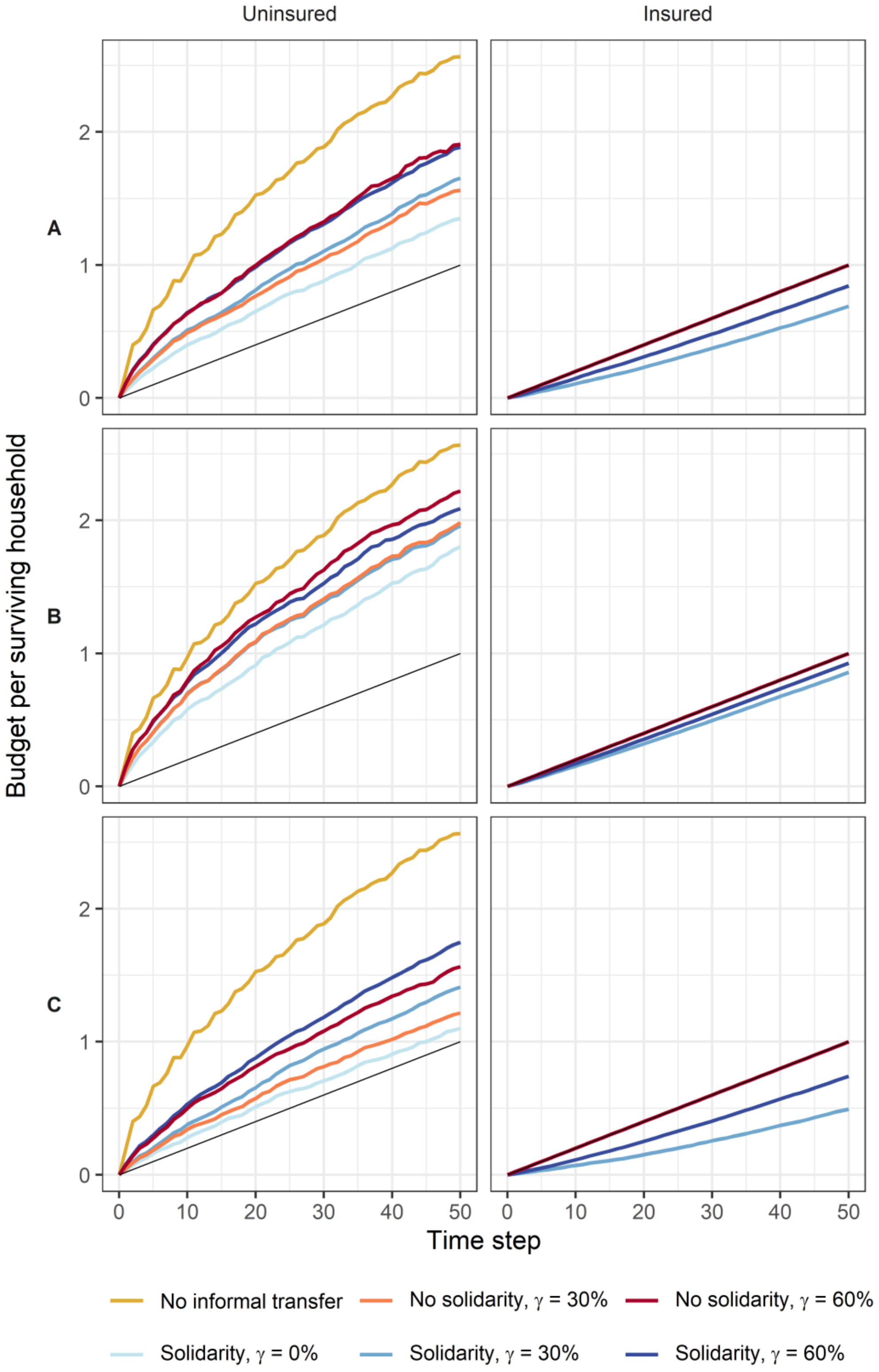

Fig S4. Budget per surviving household calculated based on the 20 households that are uninsured in every scenario and the 15 households that are insured in every investigated scenario (except $\gamma = 0\%$). The straight line shows the maximum budget that an insured household can receive as reference value. Results show (A) high rewiring probability ($N_\mathrm{N} = 4, p_\mathrm{r} = 0.8$), (B) small average degree ($N_\mathrm{N} = 2, p_\mathrm{r} = 0.2$) and (C) large average degree ($N_\mathrm{N} = 8, p_\mathrm{r} = 0.2$).
