# Supplementary material for: Informal risk-sharing between smallholders may be threatened by formal insurance: Lessons from a stylized agent-based model
Source: PLoS One. 2021 Mar 19;16(3):e0248757. doi: 10.1371/journal.pone.0248757 (PMC7978336; doi:10.1371/journal.pone.0248757)
Supplement: S4 Appendix — Additional results for idiosyncratic shocks for all economically feasible parameter with different levels of consumption and network characteristics (number of neighbors, rewiring probability). (PDF) [file pone.0248757.s004.pdf]

Additional results for idiosyncratic shocks (all parameter combinations)

To investigate the transferability of these observations to different external conditions, i.e. other levels of living costs and increased or decreased shock probability and intensity, we evaluated the status of the system for all 52 parameter combinations that were found to be economically feasible. We compared the effects of 50 years of informal transfers ($\gamma = 0\%$) on the survival rate of uninsured households to the situation 50 years after the introduction of insurance with low ($\gamma = 30\%$) and high ($\gamma = 60\%$) insurance rates, respectively. In the main text, we presented the results for a fixed income ($I = 1$) level of living costs ($C = 0.8$). Here, we show the results for lower (Fig S1) and higher (Fig S3) annual expenses. Additionally, we present the survival rates of uninsured households for a higher rewiring probability and a smaller or larger average network degree for low (Fig S1), medium (Fig S2) and high (Fig S3) level of living costs. To allow the best possible comparison between the different risk-coping instruments and insurance rates, we have again limited the analysis to the 20 households that are uninsured in the scenarios with highest insurance rate. If a panel is left blank, the parameter combination is not included in this set and therefore not selected for the analysis. Results show the mean over 100 repetitions of the number of surviving uninsured households at the last simulation step ($t = 50$).

The trends which we observed in the analysis for the selected parameter combination remains. A higher rewiring probability has also for other parameter combinations only slight effects on the survival rates of uninsured households. A smaller number of neighbors leads to lower survival rates of uninsured households, with a larger number of neighbors more uninsured households survive.

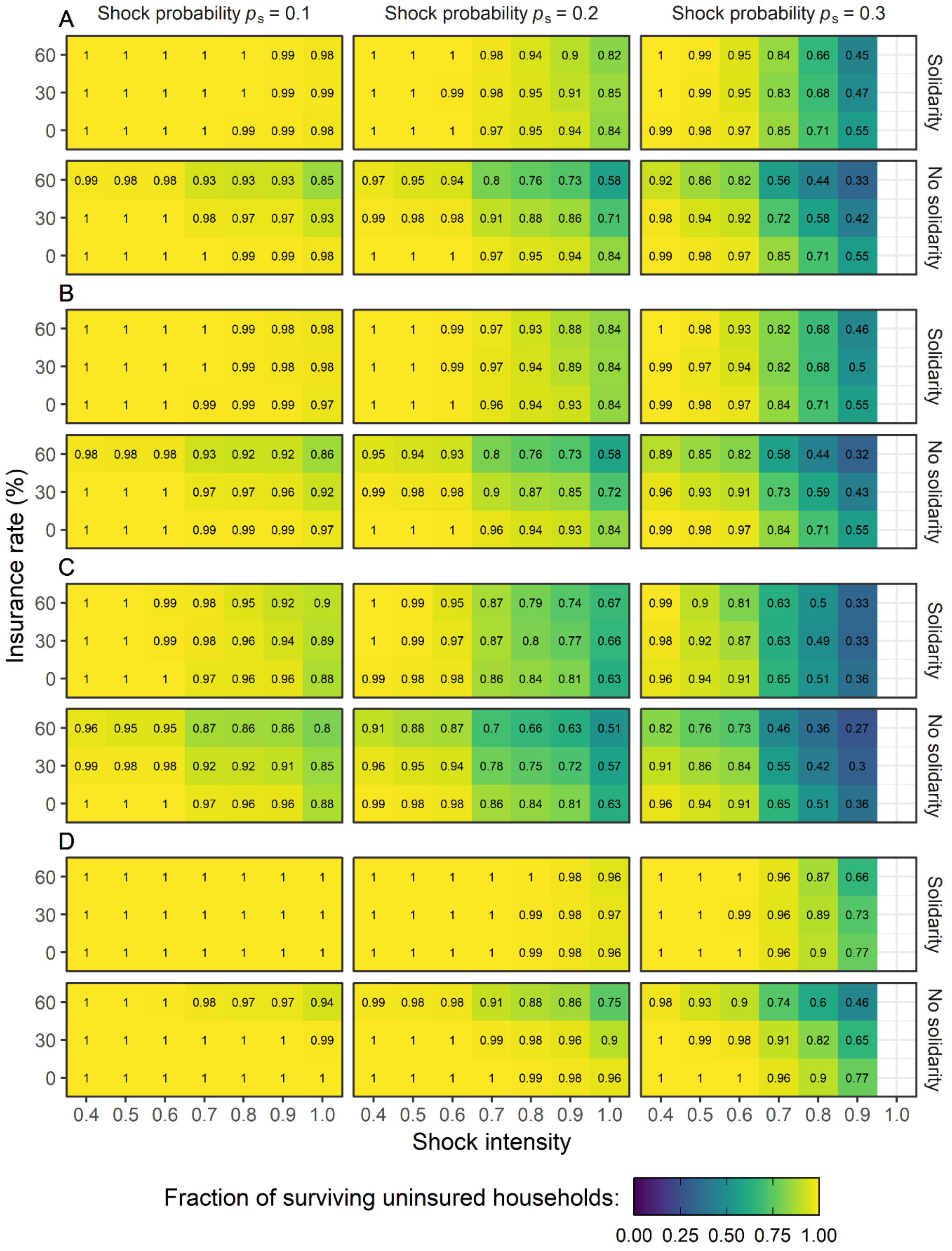

Fig S1. Fraction of surviving uninsured households among the 20 households that are uninsured in every scenario for idiosyncratic shocks and low level of living costs ($C = 0.7$) for (A) average degree and rewiring probability as in main text ($N_N = 4, p_r = 0.2$), (B) high rewiring probability ($N_N = 4, p_r = 0.8$), (C) small average degree ($N_N = 2, p_r = 0.2$) and (D) large average degree ($N_N = 8, p_r = 0.2$).

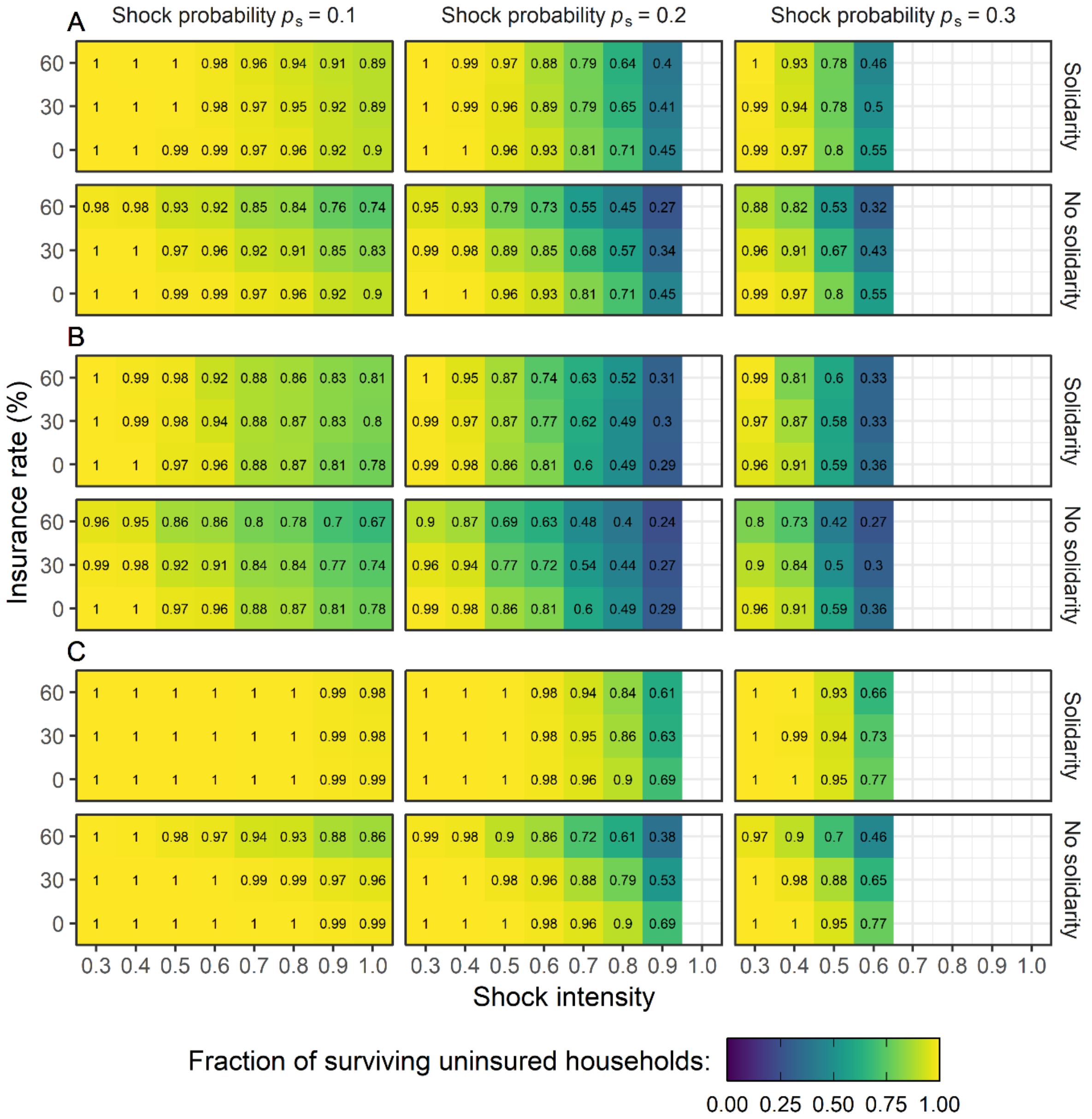

Fig S2. Fraction of surviving uninsured households among the 20 households that are uninsured in every scenario for idiosyncratic shocks and medium level of living costs ($C = 0.8$) for (A) high rewiring probability ($N_N = 4, p_r = 0.8$), (B) small average degree ($N_N = 2, p_r = 0.2$) and (C) large average degree ($N_N = 8, p_r = 0.2$).

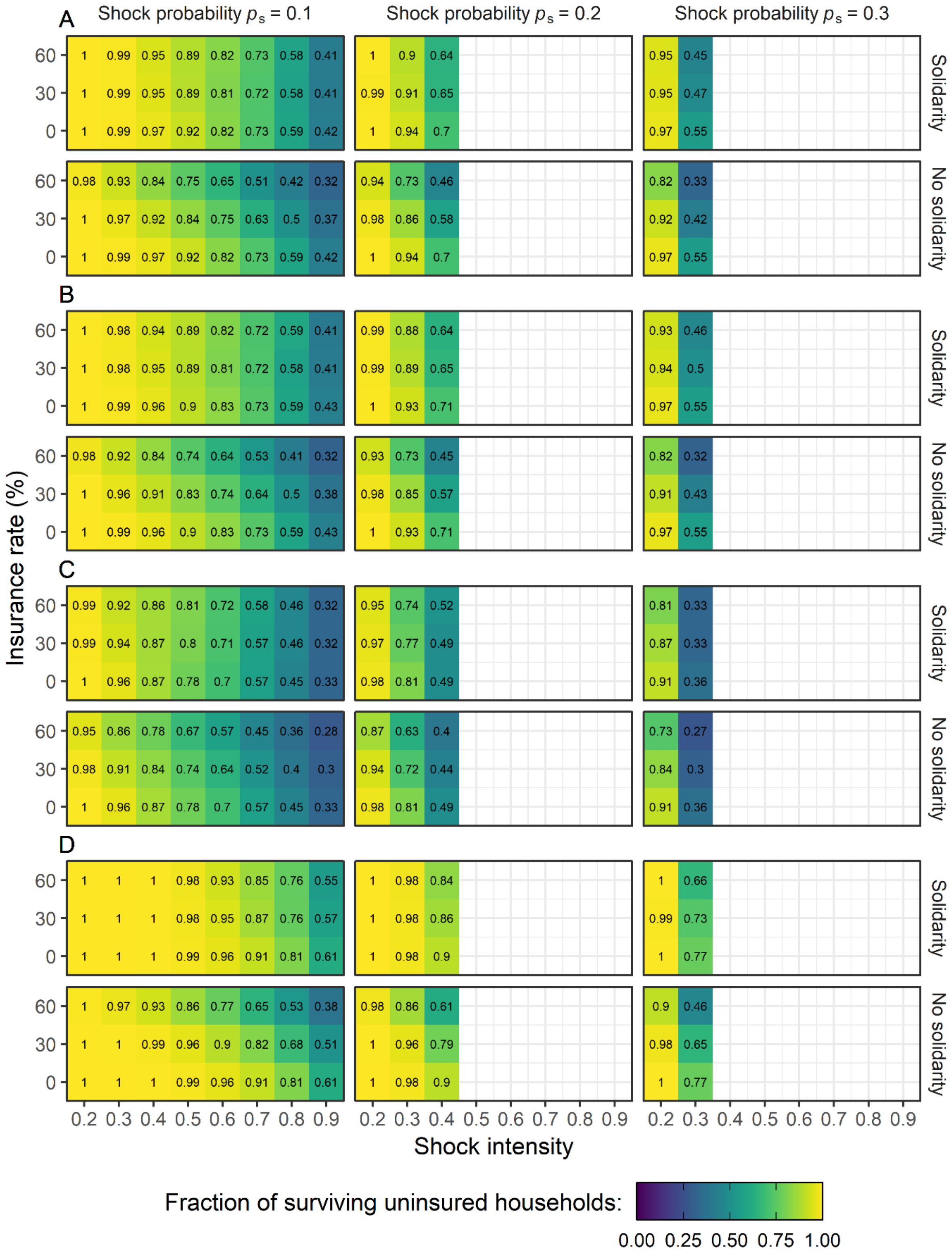

Fig S3. Fraction of surviving uninsured households among the 20 households that are uninsured in every scenario for idiosyncratic shocks and high level of living costs ($C = 0.9$) for (A) average degree and rewiring probability as in main text ($N_N = 4, p_r = 0.2$), (B) high rewiring probability ($N_N = 4, p_r = 0.8$), (C) small average degree ($N_N = 2, p_r = 0.2$) and (D) large average degree ($N_N = 8, p_r = 0.2$).
