# Supplementary material for: Informal risk-sharing between smallholders may be threatened by formal insurance: Lessons from a stylized agent-based model
Source: PLoS One. 2021 Mar 19;16(3):e0248757. doi: 10.1371/journal.pone.0248757 (PMC7978336; doi:10.1371/journal.pone.0248757)
Supplement: S5 Appendix — Additional results for covariate shocks with different levels of consumption and shock extension. (PDF) [file pone.0248757.s005.pdf]

Additional results for covariate shocks

We present additional results for covariate shocks, i.e. shock events that affect many households in a village. To make the different risk-coping instruments comparable, the order of shocks is determined at the beginning of each simulation run. Therefore, a shock series with shock probability $p_{\mathrm{V}} = p_{\mathrm{s}}/p_{\mathrm{H}}$ is created for the whole village. In time steps where the village is affected by a shock, individual households are affected with probability p_{H}. This individual shock series is equal for the same parameter combination and random seed independent of the network characteristics and risk-coping instruments that are analyzed.

In the main text, we have shown the survival rate of uninsured households for networks with average number of neighbors $N_{\mathrm{N}} = 4$ and rewiring probability $p_{\mathrm{r}} = 0.2$. Among the 52 parameter combinations that were found to be economically feasible we selected the results for a medium level of living costs ($C = 0.8$). We have presented the results for within-village shock probability $p_{\mathrm{H}} = 0.8$. In this case an individual household is affected by a shock with 80% probability if a shock event occurs at village level. Here, we additionally include the result for the more extreme case of $p_{\mathrm{H}} = 1$ where all households are affected simultaneously if a shock occurs.

We present the full parameter set divided according to the level of living costs C with $C = 0.7$ (Fig S1), $C = 0.8$ (Fig S2) and $C = 0.9$ (Fig S3). If a panel is left blank, the parameter combination is not selected for the analysis. Results show the mean over 100 repetitions of the number of surviving uninsured households at the last simulation step ($t = 50$).

We observe that, as expected, a higher within-village shock probability leads to lower survival rates of uninsured household. In this case, informal risk-coping is only possible by transfers from insured households which is not as effective as if at least some uninsured households can contribute, too. When all households are affected by shocks simultaneously and insured households do not show solidarity, the fraction of insured households has obviously no influence on the survival rate of uninsured households (lower rows in panels for $p_{\mathrm{H}} = 1$).

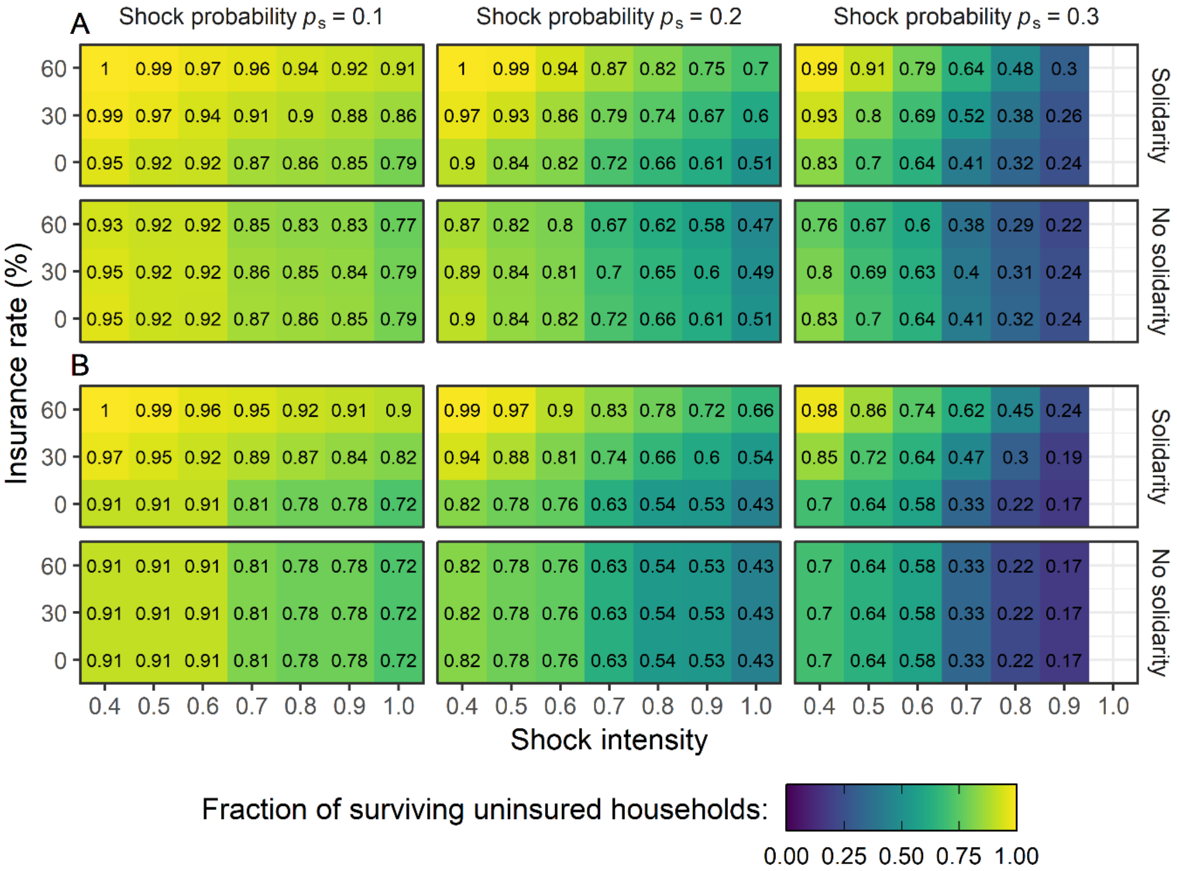

Fig S1. Fraction of surviving uninsured households among the 20 households that are uninsured in every scenario for covariate shocks ($N_{\mathrm{N}} = 4, p_{\mathrm{r}} = 0.2$) and low level of living costs ($C = 0.7$). Upper rows show the results for solidarity between all households, lower rows show the results for solidarity between uninsured households only when (A) 80% of the households ($p_{\mathrm{H}} = 0.8$) and (B) all households are affected by a shock at village level ($p_{\mathrm{H}} = 1$).

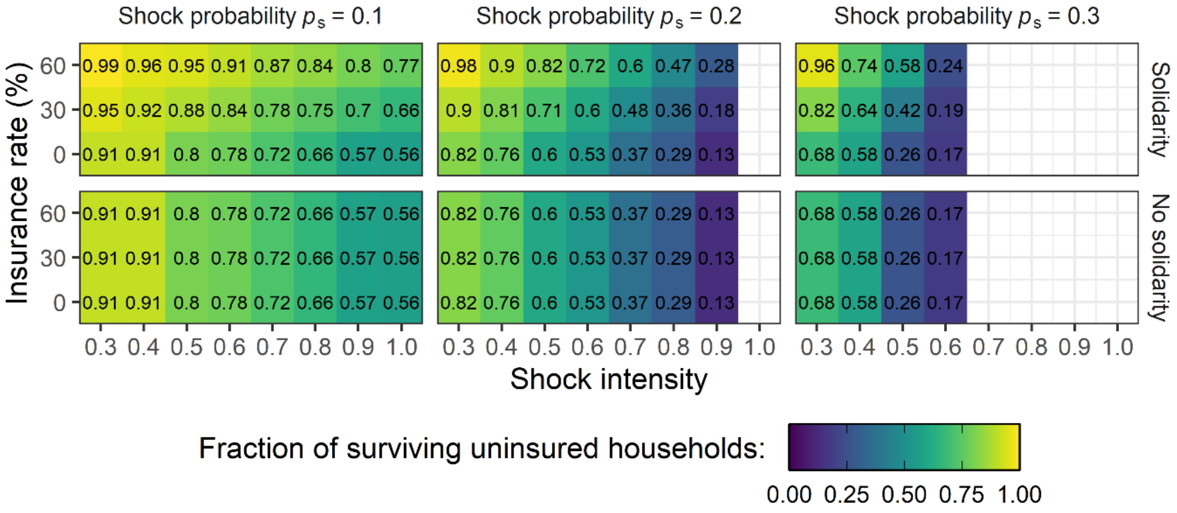

Fig S2. Fraction of surviving uninsured households among the 20 households that are uninsured in every scenario for covariate shocks ($N_{\mathrm{N}} = 4, p_{\mathrm{r}} = 0.2$) and medium level of living costs ($C = 0.8$) when all households are affected by a shock at village level ($p_{\mathrm{H}} = 1$).

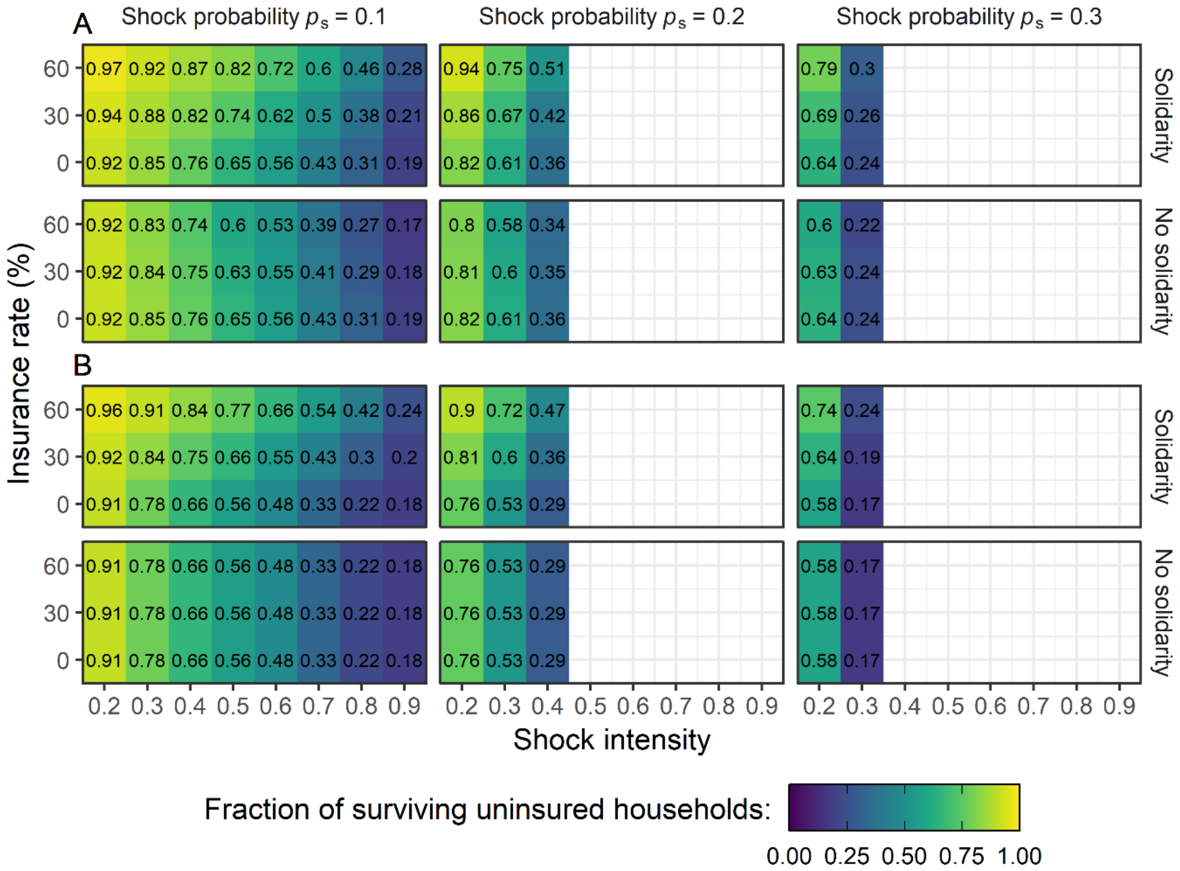

Fig S3. Fraction of surviving uninsured households among the 20 households that are uninsured in every scenario for covariate shocks ($N_N = 4, p_r = 0.2$) and high level of living costs ($C = 0.9$) when (A) 80% of the households ($p_H = 0.8$) and (B) all households are affected by a shock at village level ($p_H = 1$).
